# Supplementary material for: Combatting virulent gut bacteria by inhibiting the biosynthesis of a two-component lanthipeptide toxin
Source: Nat Commun. 2025 Jul 28;16:6936. doi: 10.1038/s41467-025-62161-7 (PMC12304293; doi:10.1038/s41467-025-62161-7)
Supplement: Supplementary file 1 — Supplementary Information [file 41467_2025_62161_MOESM1_ESM.pdf]

Supporting Information

Combatting Virulent Gut Bacteria by Inhibiting the Biosynthesis of a Two-component Lanthipeptide Toxin

Ryan Moreira[1], Bidisha Chakraborty[2], Yi Yang[1], Chandrashekhar Padhi[1], Michael S. Gilmore[3], Satish K. Nair[1,2,4,]* and Wilfred A. van der Donk[1,4,]*

[1]*Department of Chemistry and Howard Hughes Medical Institute, University of Illinois at Urbana-Champaign, Urbana, IL, 61822, USA*

[2] *Center for Biophysics and Quantitative Biology, University of Illinois at Urbana-Champaign, Urbana, IL, USA*

[3] *Departments of Ophthalmology and Microbiology, Harvard Medical School, Boston, MA 02144, USA*

[4] *Department of Biochemistry, University of Illinois at Urbana–Champaign, Urbana, IL, USA*

* Corresponding authors; snair@illinois.edu; vddonk@illinois.edu

Supplementary Figure 1. High resolution mass spectra of compounds **1-5**	S3
Supplementary Figure 2. High resolution mass spectra of compounds **6-10**	S4
Supplementary Table 1: Analysis of the high-resolution masses observed for compounds **1-10**	S5
Supplementary Table 2. UPLC retention times of **1-10**	S5
Supplementary Table 3. Primers used for RT-qPCR analysis *E. faecalis* ATCC 29212	S6
Supplementary Table 4. Data collection and refinement statistics	S7
Supplementary Figure 3. Measuring cytolysin maturation by CylA using a pyranine pore formation assay	S8-S11
Supplementary Figure 4. High-resolution MS-MS analysis of the FRET peptide	S12
Supplementary Figure 5. The FRET peptide is cleaved by CylA on the C-terminal side of the Glu residue producing two fragments	S13
Supplementary Figure 6. Pre-cytolysin peptides can activate CylA	S14
Supplementary Figure 7. Full length analytical SEC traces	S15
Supplementary Figure 8. SDS-PAGE analysis of fractions collected during analytical SEC of a sample of CylA	S16

Supplementary Figure 9. Treatment of samples of His6-CylA-27-412 with substrate reduces the amount of CylA-96-412◦His6-27-95 relative to CylA-96-412.	S17
Supplementary Figure 10. Circular dichroism trace of the pro-domain of CylA	S18
Supplementary Figure 11. Experimentally determined structure of CylA-96-412◦His6-27-95 superimposed on the predicted structure generated using AlphaFold 3	S18
Supplementary Figure 12. Surface representation of CylA-96-412◦His6-27-95 showing the extensive interactions between the pro-domain and the catalytic domain	S19
Supplementary Figure 13. Two-dimensional interaction diagram showing the interactions between the pro-domain cleavage peptide and active site residues in the catalytic domain.	S20
Supplementary Figure 14. Hypothetical model of compound **9** in a covalent complex with Ser359 of the CylA-96-412◦His6-27-95 active site.	S21
Supplementary Figure 15. Alignment of the C-terminal domain of CylA from the crystal structure with the same region from the AlphaFold 3 structure of CylA:GDVQAE	S22
Supplementary Figure 16. Overlay of the pro-domain of CylA, the RRE of NisB and NHLP type LPs found in the proteusins.	S23
Supplementary Figure 17. Inhibitor 9 did not affect the growth of *E. faecalis* FA2-2 (pAM714) or *E. faecalis* ATCC 29212.	S24
Supplementary Figure 18. Cytotoxicity of boronic acid inhibitors **2**, **3**, **5-9** against HeLa cells.	S25
Supplementary Figure 19. Stability of inhibitor **9**	S26
Supplementary Figure 20. ^{13}C NMR spectrum of **15**	S27
Supplementary Figure 21. ^{1}H NMR spectrum of **15**	S28
Supplementary Figure 22. Analytical UPLC analysis of purified **1-10**	S29-S33
References	S34

Primary data associated with this study can be found at:

Moreira, Ryan; van der Donk, Wilfred (2025), “Data associated with "Combatting Virulent Gut Bacteria by Inhibiting the Biosynthesis of a Two-component Lanthipeptide Toxin"”, Mendeley Data, V1, doi: 10.17632/yh5kp2fzjn.1

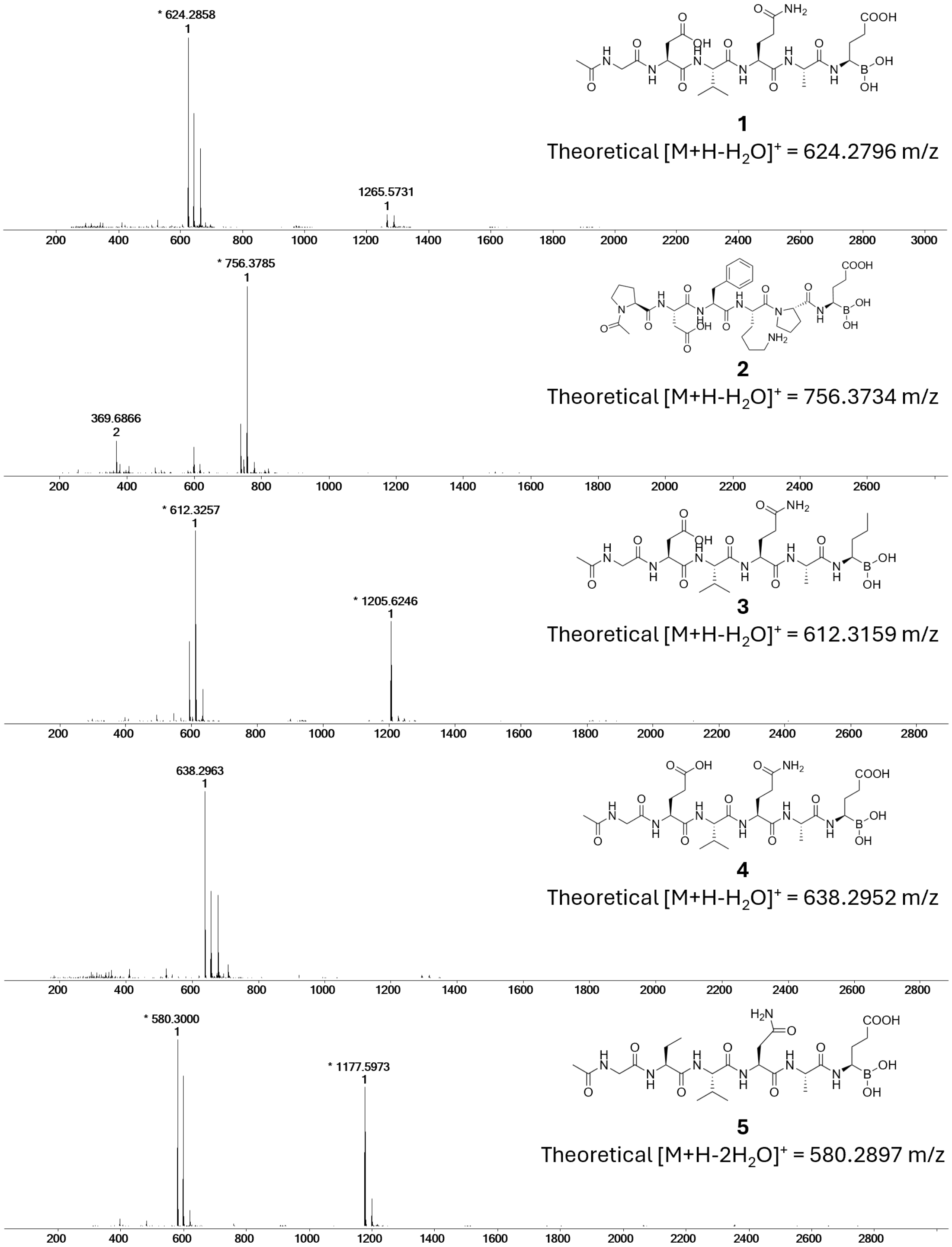

Supplementary Figure 1. High resolution mass spectra of compounds **1**-**5**. Peaks are labelled with m/z values and charge states denoted below the m/z values. Source data are provided as a Source Data file. For assignments of observed masses, see Supplementary Table 1.

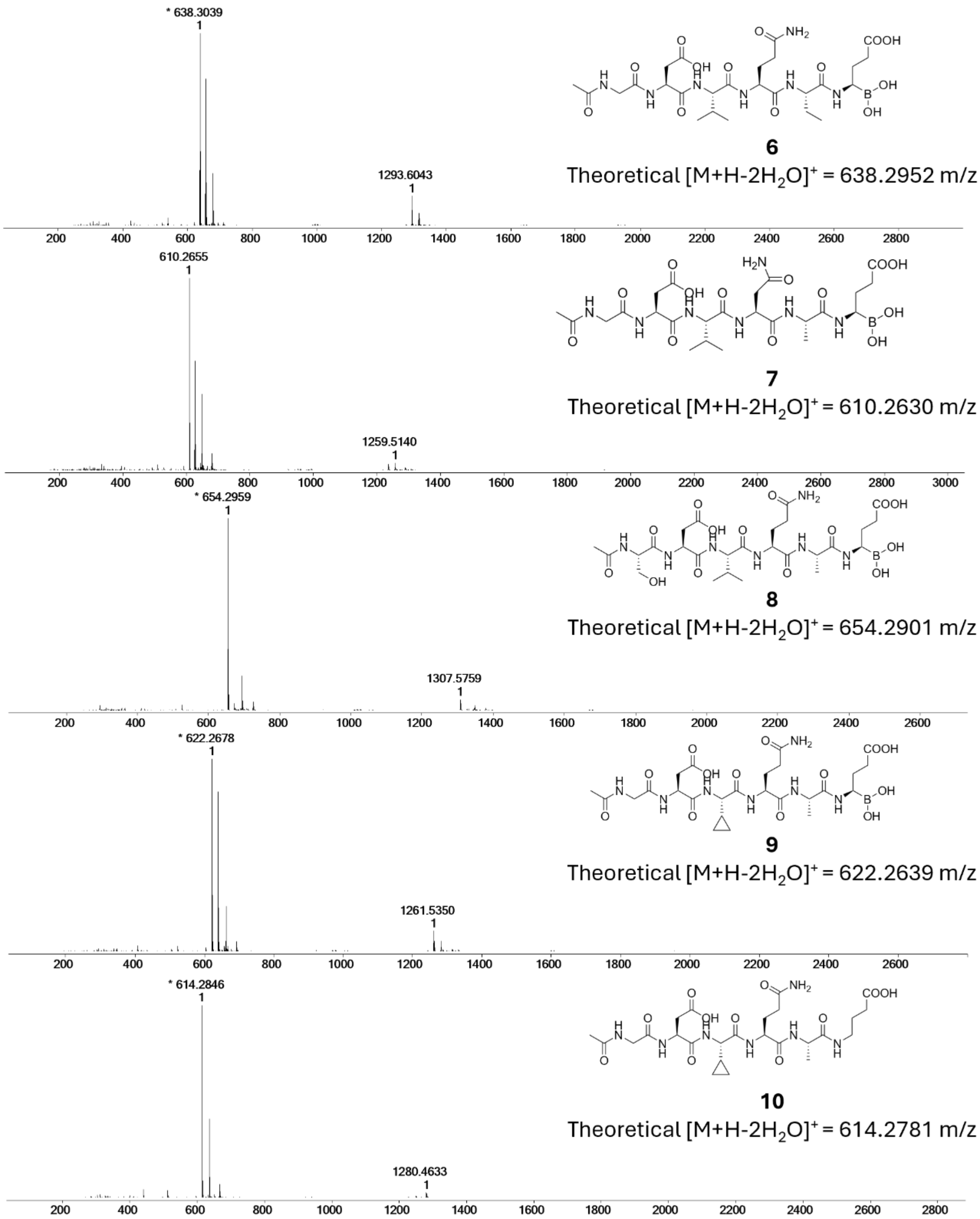

Supplementary Figure 2. High resolution mass spectra of compounds **6-10**. Peaks are labelled with m/z values and charge states denoted below the m/z values. Source data are provided as a Source Data file. For assignments of observed masses, see Supplementary Table 1.

Supplementary Table 1. Analysis of the high-resolution masses observed for compounds **1-10**

Compound number	Ion	Theoretical m/z	Observed m/z	Δ (ppm)
**1**	$[M+H-H_2O]^+$	624.2796	624.2858	6.2
**1**	$[2M+H-3H_2O]^+$	1265.5623	1265.5639	1.6
**2**	$[M+H-H_2O]^+$	756.3734	756.3776	4.2
**2**	$[M+2H-2H_2O]^+$	369.6851	369.6863	1.2
**3**	$[M+H-H_2O]^+$	612.3159	612.3236	7.7
**3**	$[2M+H-3H_2O]^+$	1205.6140	1205.6190	5.0
**4**	$[M+H-2H_2O]^+$	638.2952	638.2968	1.6
**5**	$[M+H-2H_2O]^+$	580.2897	580.3000	10.3
**5**	$[2M+H-3H_2O]^+$	1177.5827	1177.5924	9.7
**6**	$[M+H-2H_2O]^+$	638.2952	638.3034	8.2
**6**	$[M+H-3H_2O]^+$	1293.5937	1293.6039	10.2
**7**	$[M+H-2H_2O]^+$	610.263	610.2670	4.0
**7**	$[2M+H-3H_2O]^+$	1237.5311	1237.5351	4.0
**8**	$[M+H-2H_2O]^+$	654.2901	654.2959	5.8
**8**	$[M+H-4H_2O]^+$	1307.5759	1307.5729	3.0
**9**	$[M+H-2H_2O]^+$	622.2639	622.2678	3.9
**9**	$[2M+H-3H_2O]^+$	1261.5311	1261.5350	3.9
**10**	$[M+H]^+$	614.2781	614.2841	6.0

Supplementary Table 2. UPLC retention times of **1-10**

Compound Number	Retention time (min)*
**1**	5.33
**2**	5.33
**3**	5.62
**4**	5.43
**5**	5.93
**6**	5.89
**7**	4.51
**8**	4.98
**9**	3.70
**10**	5.15

*Determined using Agilent 1290 LC-MS QToF instrument equipped with a Poroshell C18 2.7 µm 120 Å 100 x 3.0 mm column. The following LC method was used for separation: 98% H_2O + 0.1% formic acid (FA, solvent A)/2% CH_3CN + 0.1% FA (solvent B) for 2 min then a linear gradient from 98:2 to 2:98 solvent A:solvent B over 6 min.

Supplementary Table 3. Primers used for RT-qPCR analysis *E. faecalis* ATCC 29212

Gene	Primer	Sequence
E. faecalis 16S rRNA	FP	CGCTTCTTTCCTCCCGAGT
	RP	GCCATGCGGCATAAACTG
cylL	FP	CTGTTGCGGCGACAGCT
	RP	CCACCAACCCAGCCACAA
cylS	FP	GTGCTAAATAAGGAAAATCAAGAAAACTATTACTC
	RP	CAAAAGAAGGACCAACAAGTTCTAATT
cylA	FP	TGACTCGGGGATTGATAGGC
	RP	TGTCCCATCCATCACCTTGT

Supplementary Table 4. Data collection and refinement statistics

	CylA pro and catalytic domains
**Data collection**	
Wavelength (Å)	0.9686
Space group	C2
Unit Cell (Å/degrees)	128.44, 56.92, 50.82/ 102.47
Resolution range (Å)[1]	28.5 - 1.3 (1.3 -1.304)
Total reflections	493,204
Unique reflections	80,018
Multiplicity	6.2 (4.6)
Completeness (%)	91.0 (51.7)
Mean I/sigma (I)	23.5 (2.5)
R-merge (%)[2]	3.8 (62.1)
R-pim (%)	1.6 (31.9)
CC ½	1.000 (0.802)
**Refinement**	
Resolution (Å)	25.0-1.3
Number of reflections	79,789
R-work	17.1
R-free[3]	18.6
Number of atoms	
Macromolecules	2,938
Solvent	471
Average B-factor	
Macromolecules	19.6
Solvent	33
RMS (bond lengths)	0.006
RMS (bond angles)	1.376
Molprobity clash score	5.46
Favored (%)	97.3
Allowed (%)	2.7
Outliers (%)	0.0

1. Highest resolution shell is shown in parenthesis.
2. $R_{merge} = \Sigma |(I_i - \langle I_i \rangle | \Sigma I_i$ where I_i = intensity of the *i*th reflection and $\langle I_i \rangle$ = mean intensity.
3. R-factor = $\Sigma(|F_{obs}|-k|F_{calc}|)/\Sigma |F_{obs}|$ and R-free is the R value for a test set of reflections consisting of a random 5% of the diffraction data not used in refinement.

Compound **1**:

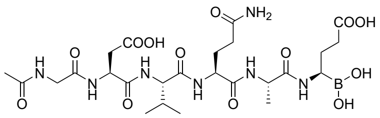

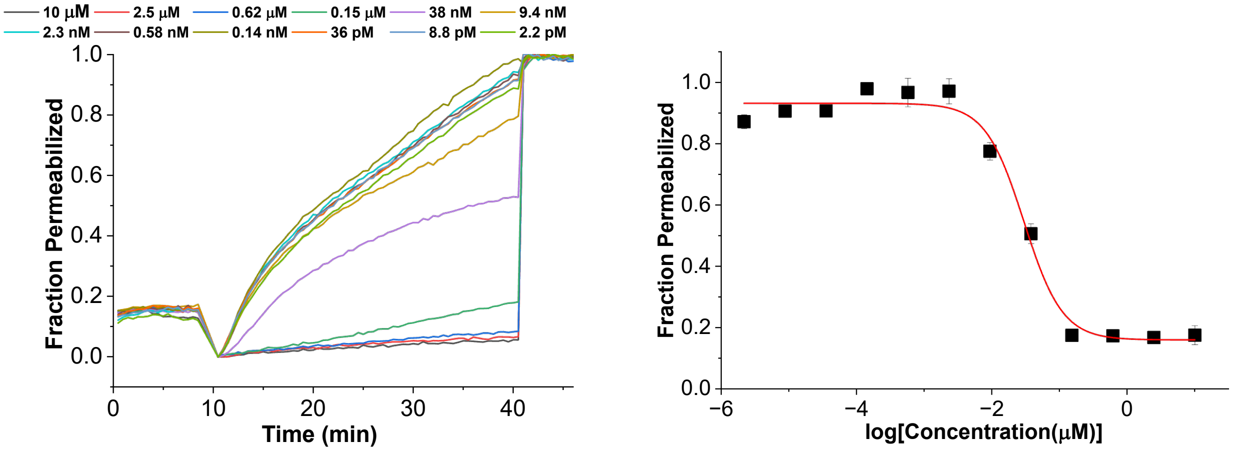

Compound **2**:

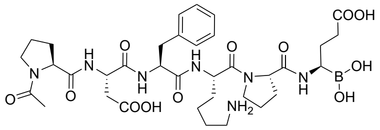

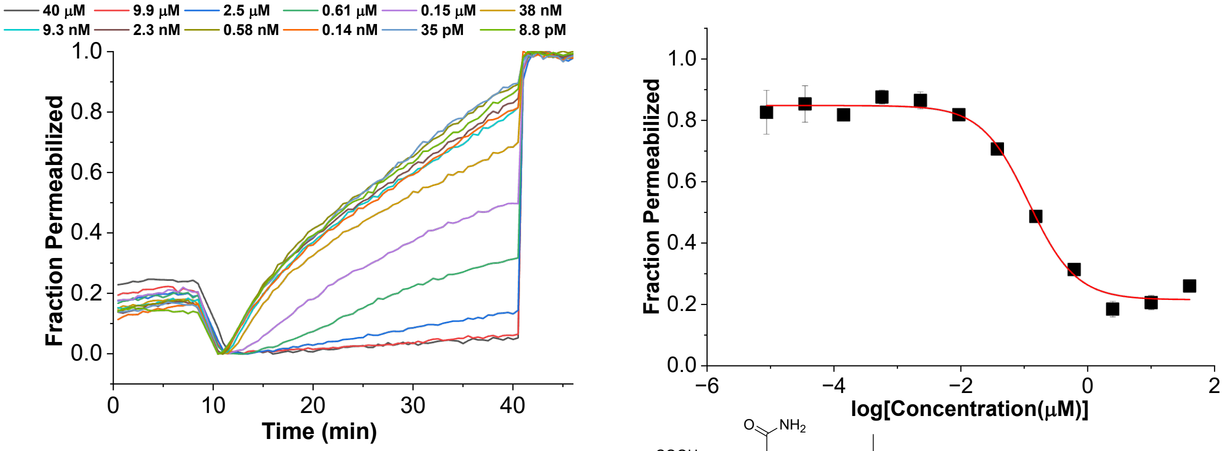

Compound **3**:

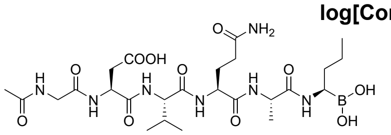

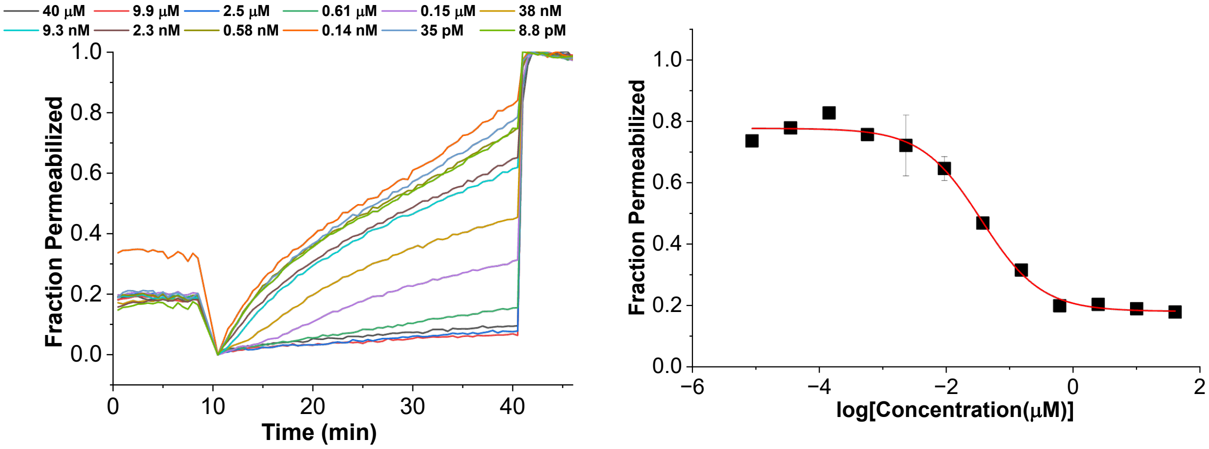

Compound **4**:

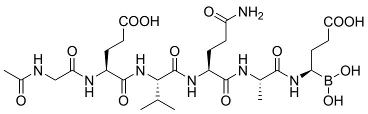

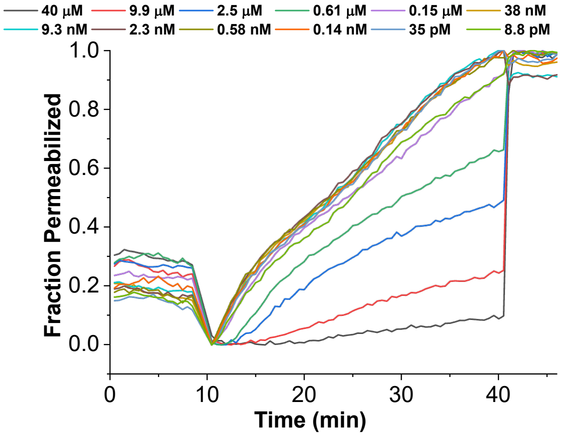

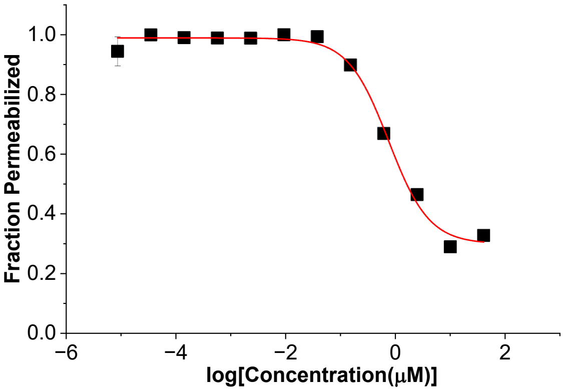

Compound **5**:

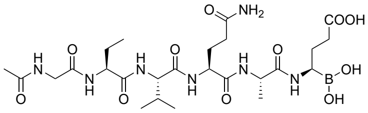

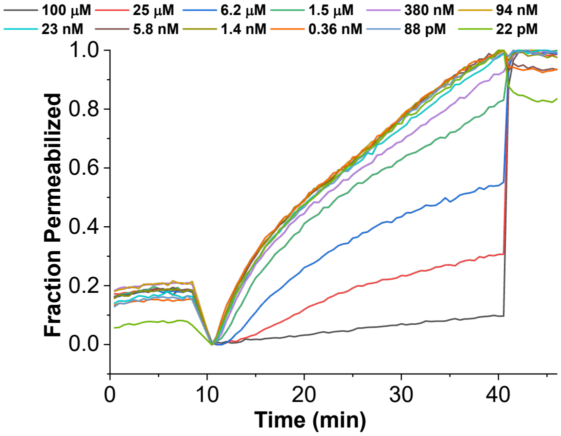

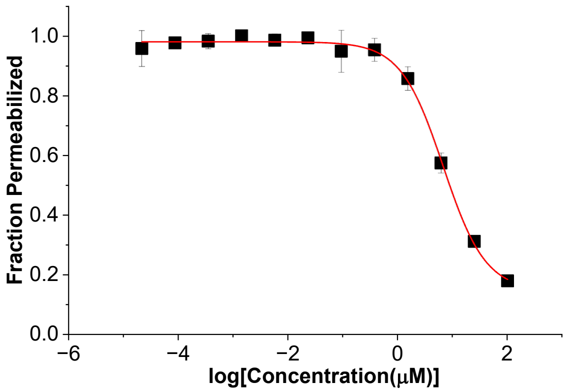

Compound **6**:

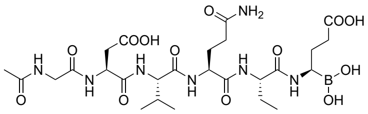

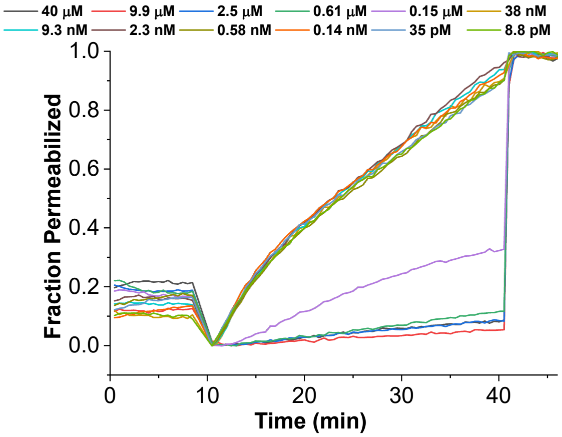

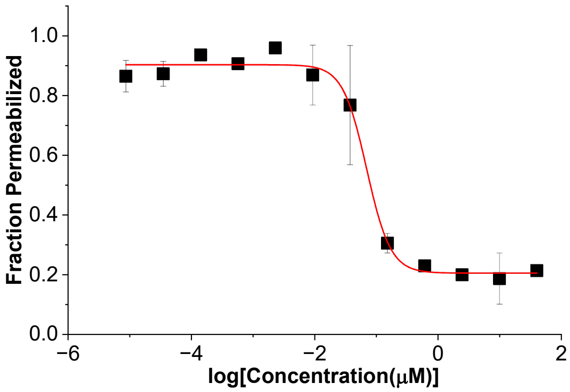

Compound **7**: 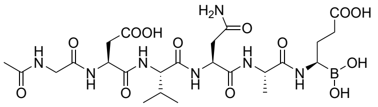

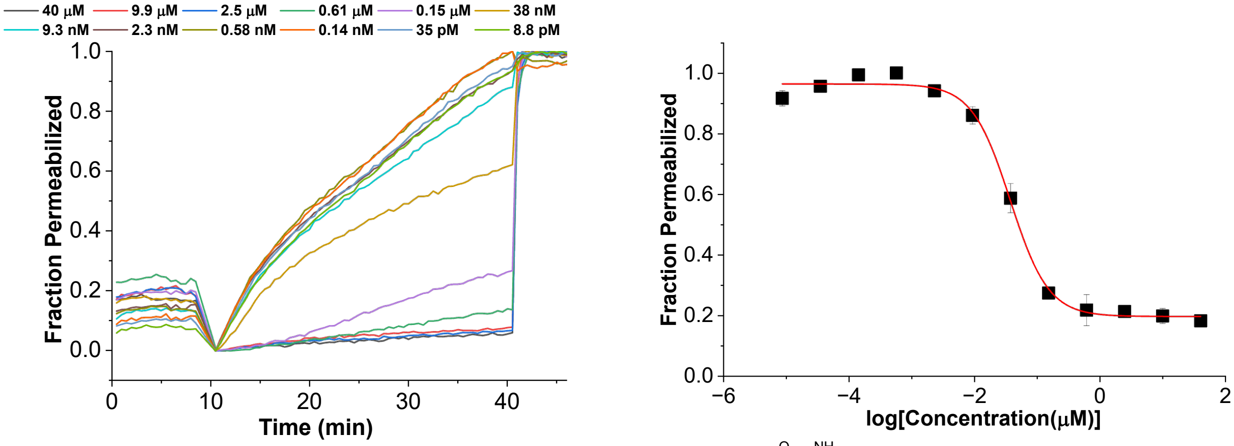

Compound **8**: 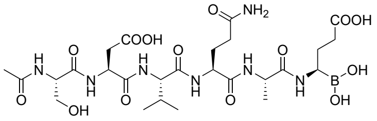

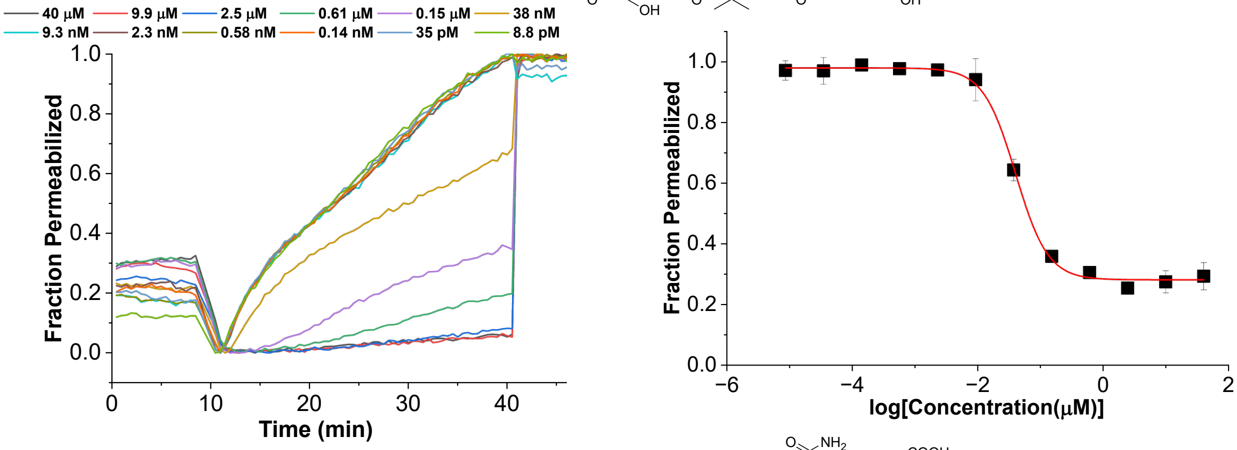

Compound **9**: 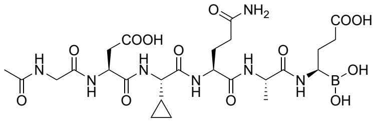

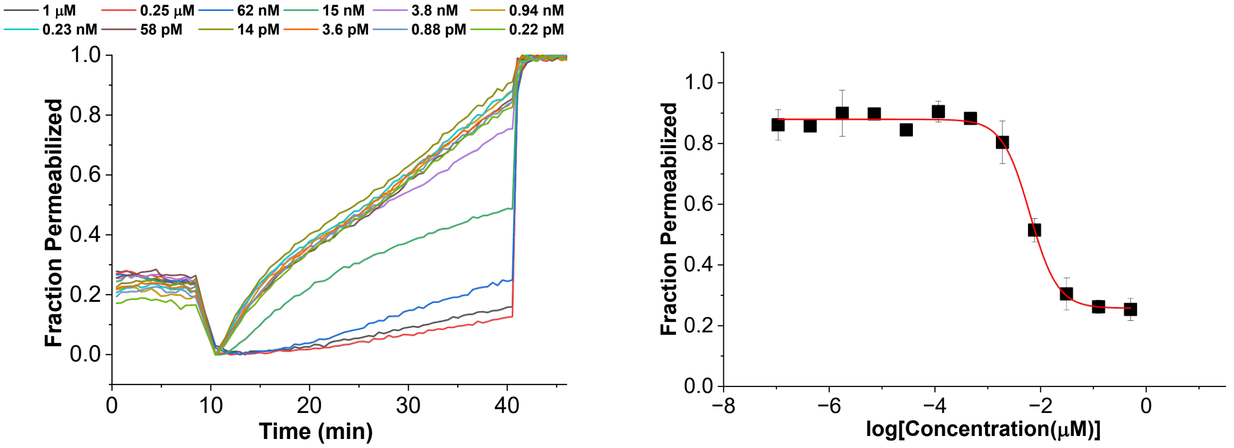

Compound **10**:

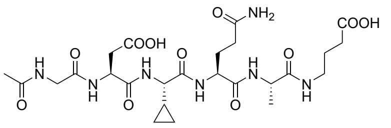

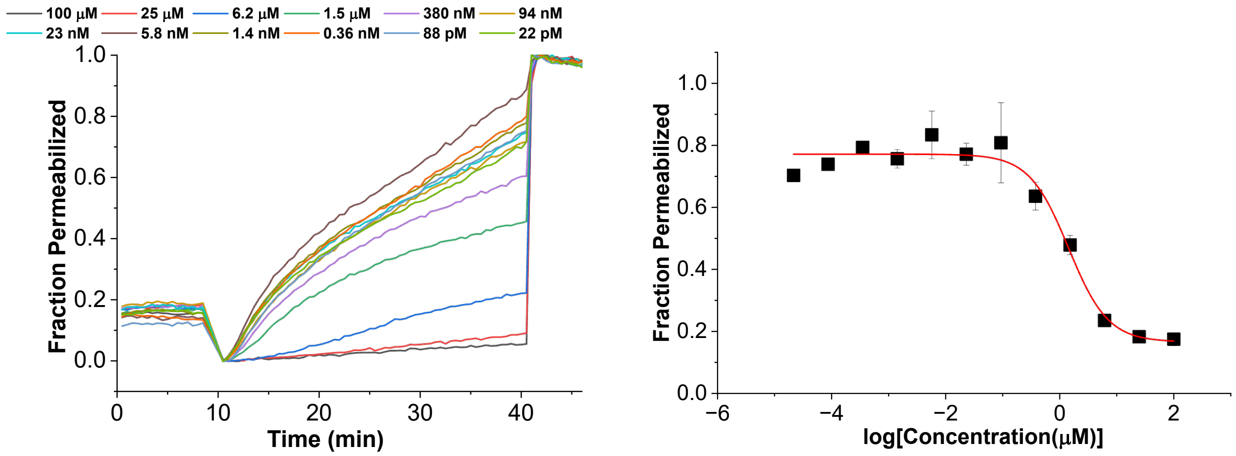

Supplementary Figure 3. Measuring CylA-catalyzed cytolysin maturation using a pyranine pore formation assay. Plots on the left side show the normalized pyranine emission intensity monitored continuously over time. A 1:1 mixture of $mCylL_S$:$mCylL_L$ was added after 10 min. Triton X-100 was added 30 min later. Each trace was collected using a different total concentration of inhibitor as indicated in the legend. Traces are from a single representative trial. On the right side, the fraction of liposomes permeabilized after 30 min was plotted as a function of total inhibitor concentration. The data were fit to a dose-response function using Origin 7 and the fit curve is depicted by the red line. Errors bars represent the standard of deviation between two replicates (*n* = 2). Source data are provided as a Source Data file.

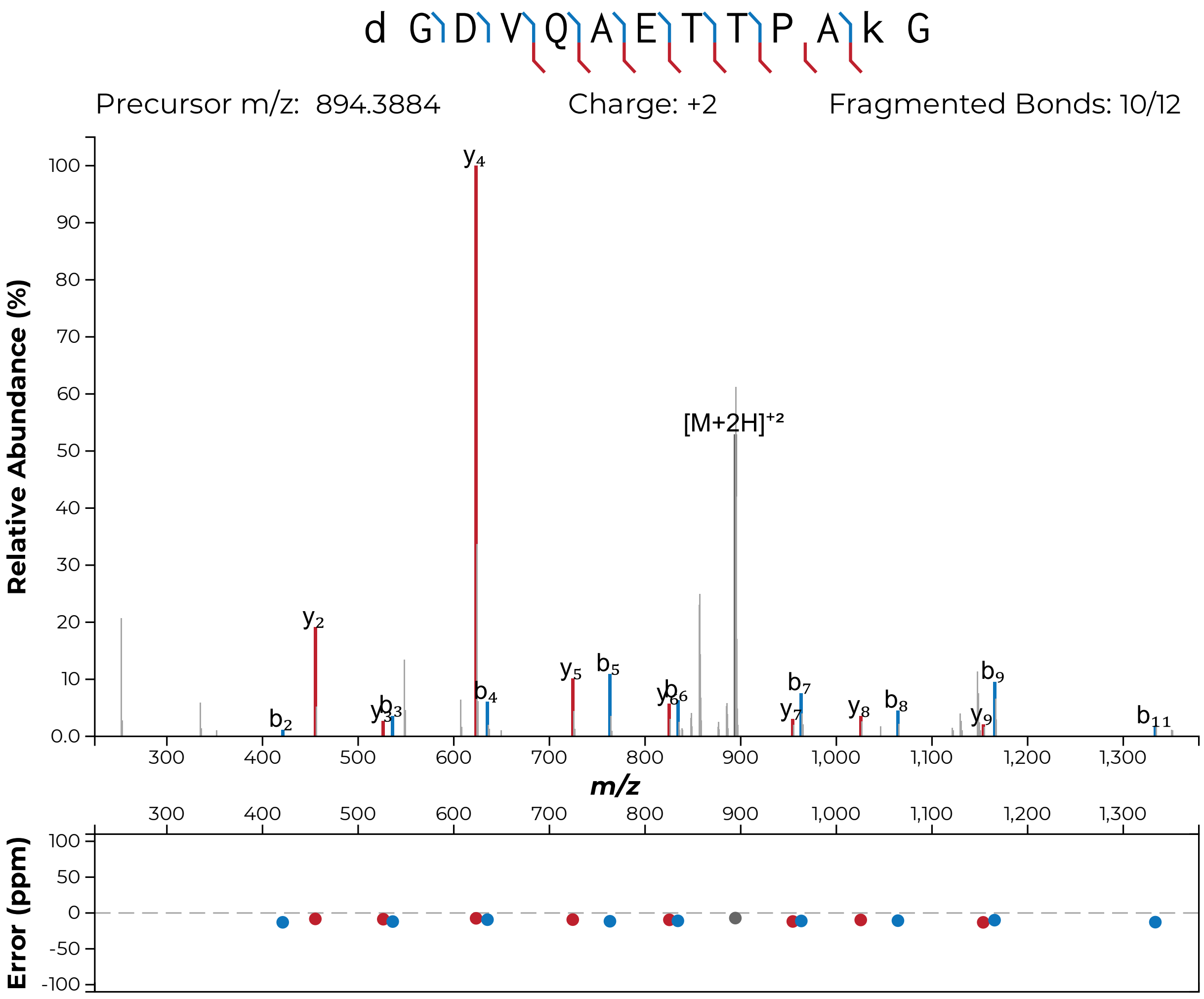

Supplementary Figure 4. High-resolution MS-MS analysis of the FRET peptide. Residues represented by lower-case letters are modified at the side chain. 'd' bears the EDANS moiety and 'k' bears the DABCYL moiety. Source data are provided as a Source Data file. Figure prepared using the Interactive Peptide Annotator Webtool[1].

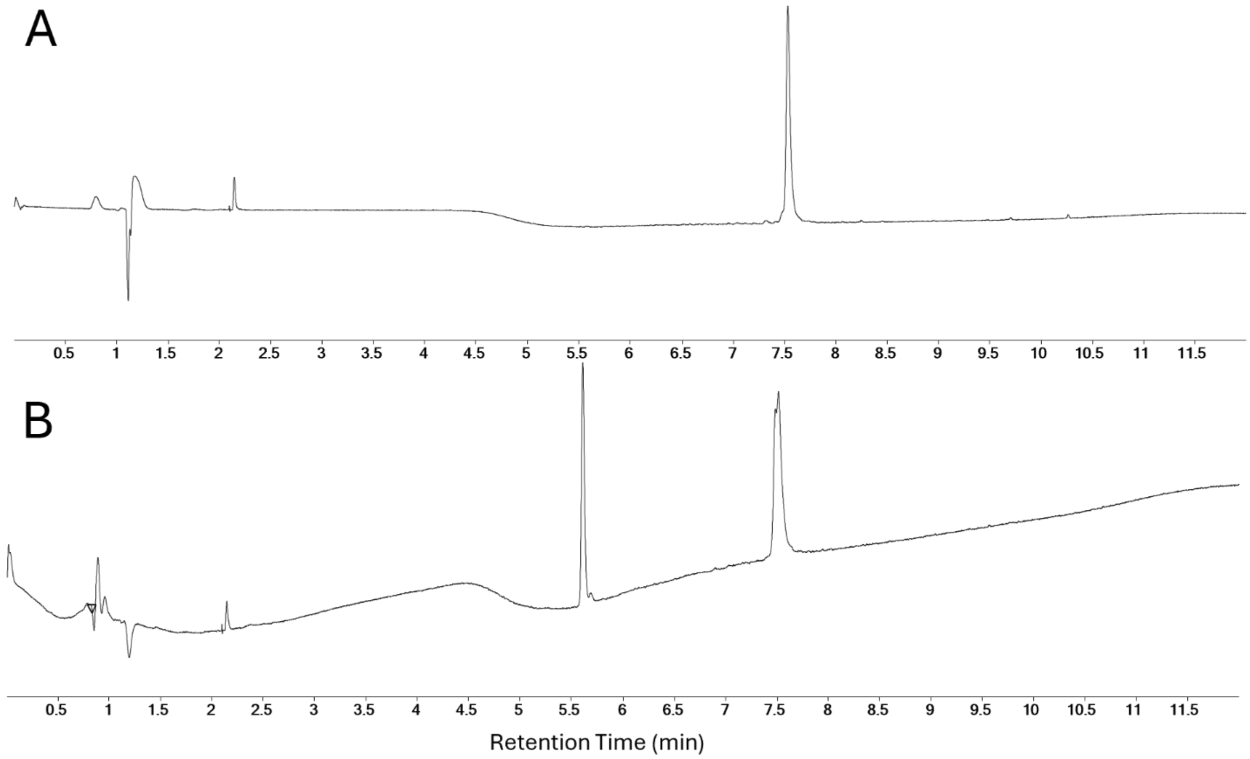

Supplementary Figure 5. The FRET peptide is cleaved by CylA on the C-terminal side of the Glu residue producing two fragments. A) A chromatogram of a sample of untreated FRET peptide. B) A chromatogram of a sample of FRET peptide incubated with CylA in PBS (pH = 7.4) overnight at 37℃. The N-terminal fragment has a retention time equal to 5.61 min and a high-resolution mass-to-charge ratio equal to 981.3518 m/z corresponding to [M+H] which has a theoretical mass equal to 981.3618. The C-terminal fragment has a retention time equal to 7.52 min and a high-resolution mass-to-charge ratio equal to 825.4179 corresponding to [M+H], which has a theoretical mass equal to 825.4254. Both traces represent the absorbance at 340 nm over the course of the LC run. Detailed conditions are provided in the Methods section of the manuscript. Source data are provided as a Source Data file.

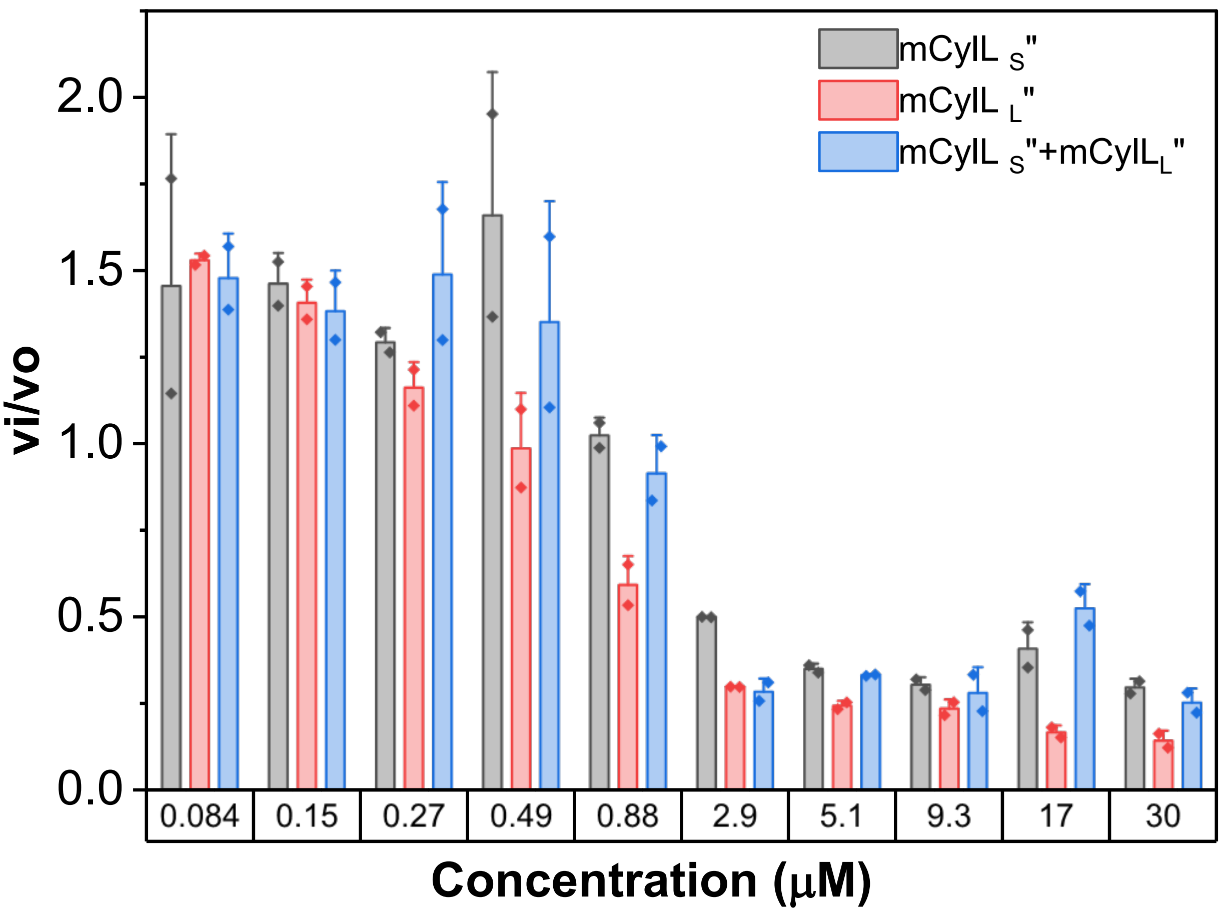

Supplementary Figure 6. Pre-cytolysin peptides can activate CylA. The pre-cytolysin peptides $mCylL_L$, $mCylL_S$ and a 1:1 mixture of $mCylL_L$:$mCylL_S$ increase the activity of CylA at concentrations less than 1 μM. Initial rates were measured as described in the 'Determining initial rates using the FRET peptide substrate' section of the Methods. Relative enzyme activity (v_i/v_o) was calculated by dividing the initial velocity of the proteolysis reaction in the presence of pre-cytolysin peptides (v_i) by the velocity observed in the absence of pre-cytolysin peptides (v_o). In wells free of pre-cytolysin peptides, v_i/v_o is equal to 1. Error bars represent the standard of deviation (n = 2). Source data are provided as a Source Data file.

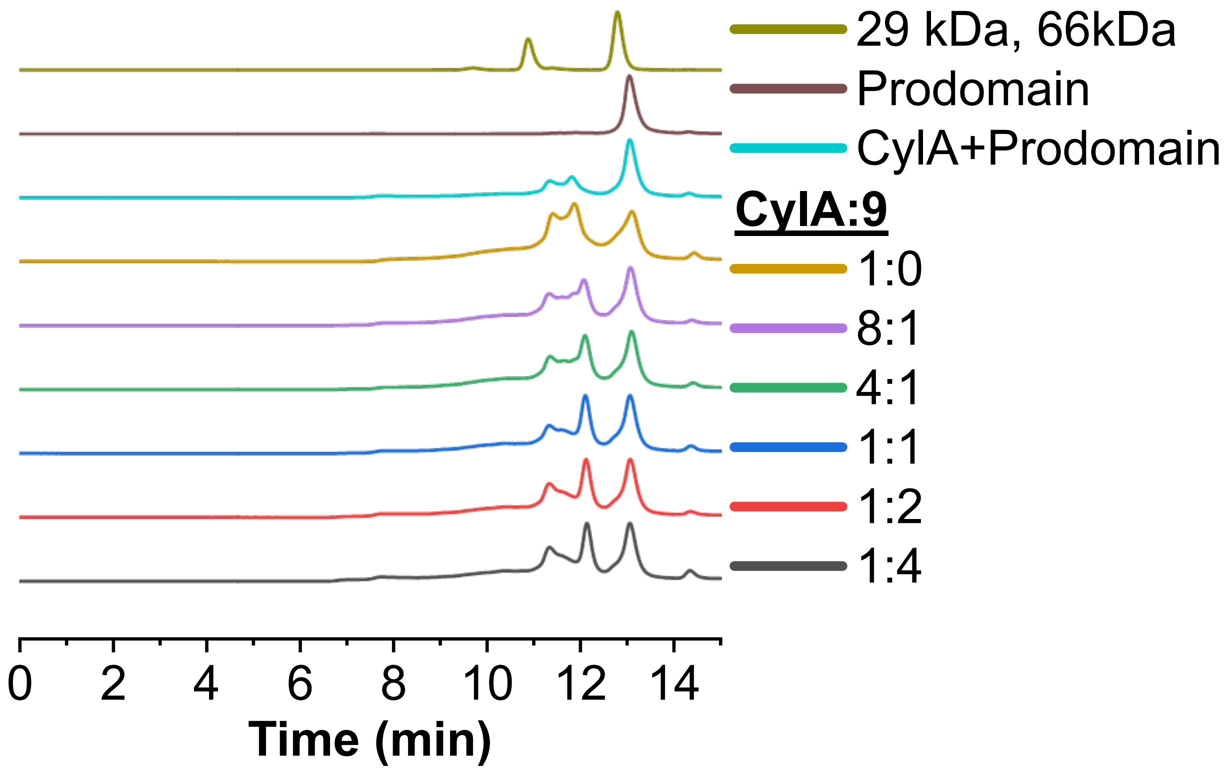

Supplementary Figure 7. Full length analytical SEC traces. The first trace from the top is a mixture of carbonic anhydrase from bovine erythrocytes (29 kDa) and BSA (66 kDa). The second trace is a sample of SEC purified pro-domain. The third trace is the full chromatogram of the pro-domain-CylA mixture featured in Fig. 5E. The remaining traces are the full-length versions of the chromatograms featured in Fig. 5D. Source data are provided as a Source Data file.

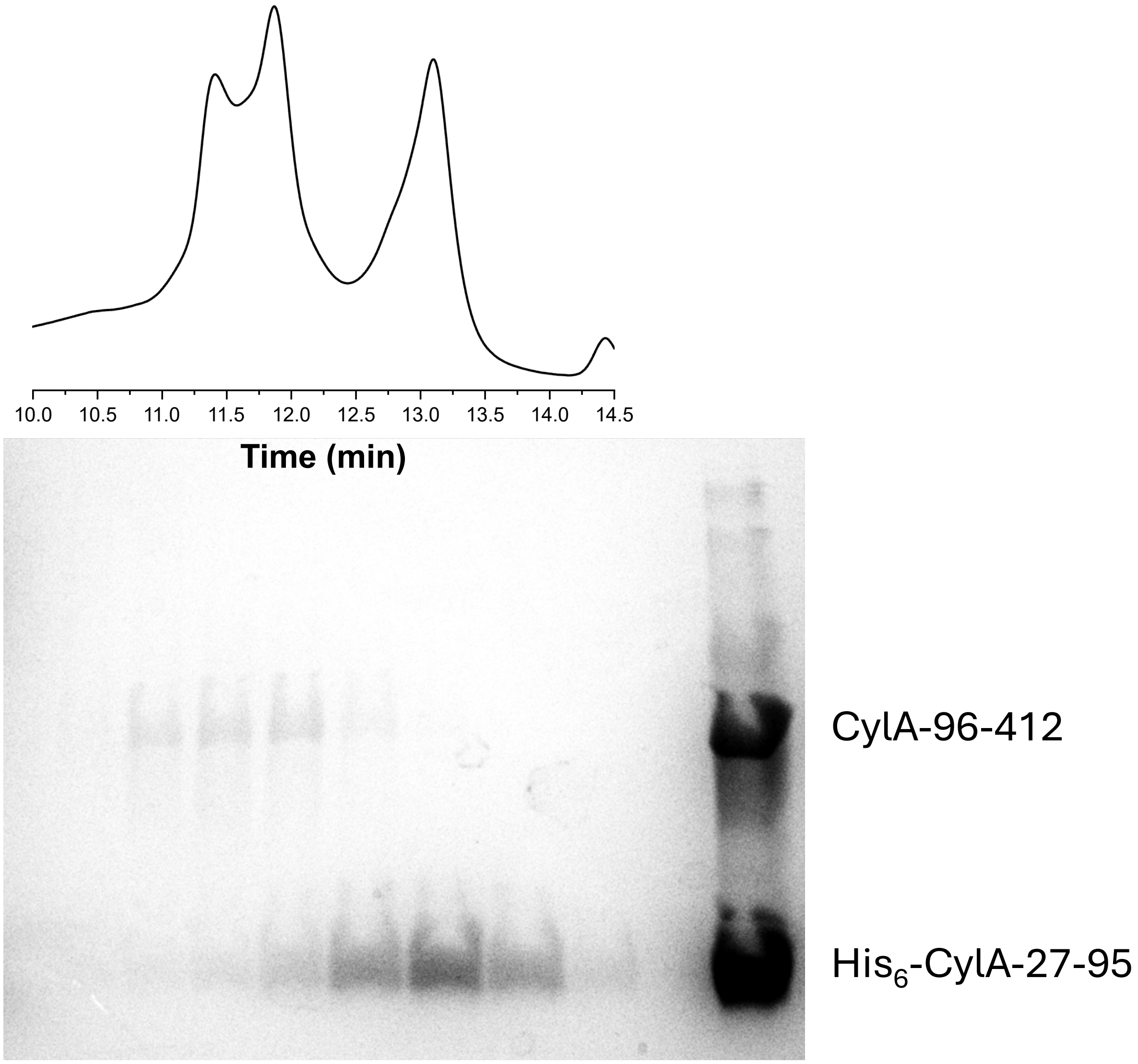

Supplementary Figure 8. SDS-PAGE analysis of fractions collected during analytical SEC of a sample of CylA. The chromatogram is shown above the gel image and the retention time is aligned with the lanes on the gel. Fractions were changed every 30 s starting at the 10 min retention time.

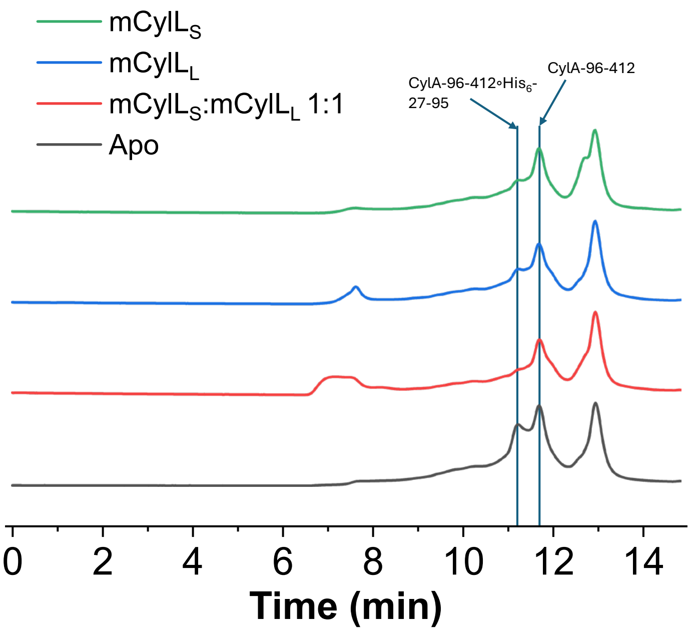

Supplementary Figure 9. Treatment of samples of His$_6$-CylA-27-412 with substrate reduces the amount of CylA-96-412∘His$_6$-27-95 relative to CylA-96-412. SEC purified His$_6$-CylA-27-412 was diluted to 50 μM and combined with 1 equivalent of one of the following: mCylL$_S$, mCylL$_L$, or mCylL$_S$:mCylL$_L$ (1:1). After incubation for 10 min at room temperature, the sample was analyzed by analytical SEC. Source data are provided as a Source Data file.

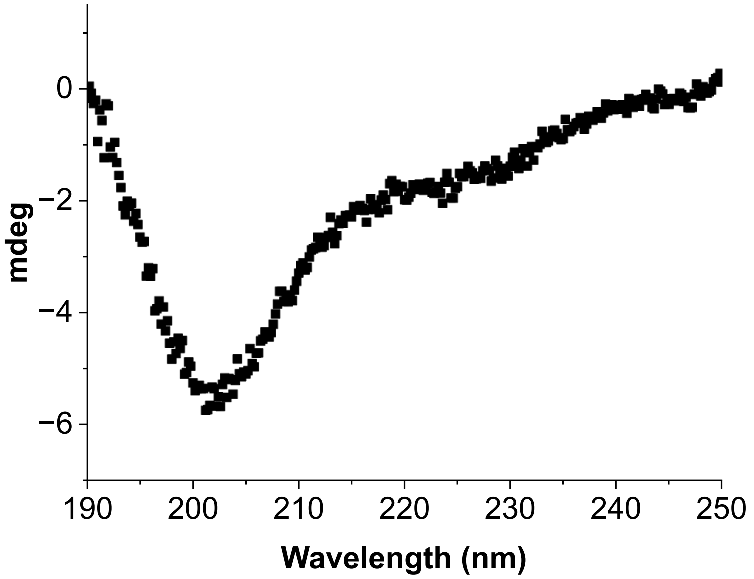

Supplementary Figure 10. Circular dichroism trace of His_6-CylA-27-95 isolated during SEC purification of CylA. Source data are provided as a Source Data file.

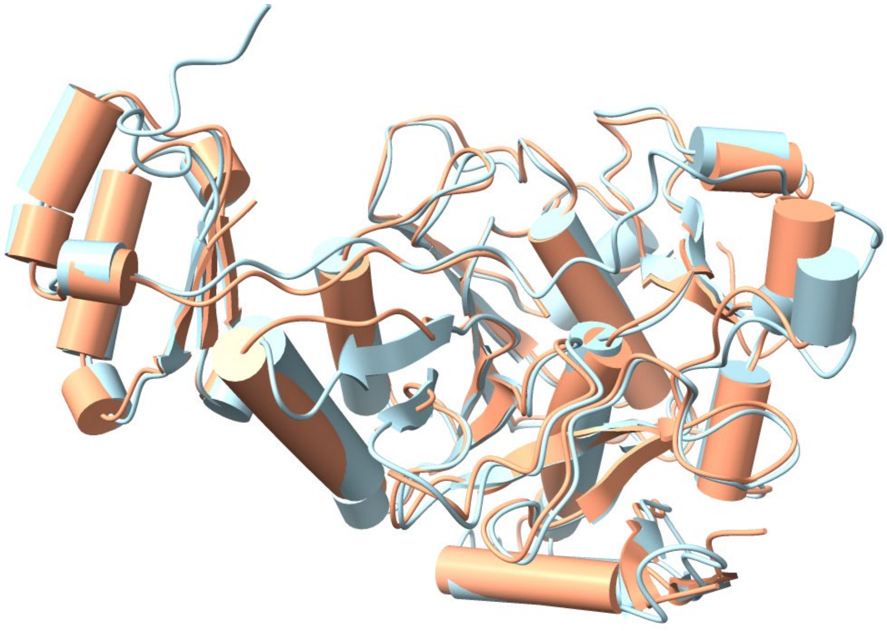

Supplementary Figure 11. Experimentally determined structure of CylA-96-412◦His_6-27-95 (tan) superimposed on the predicted structure (cyan) generated using Alphafold3[2].

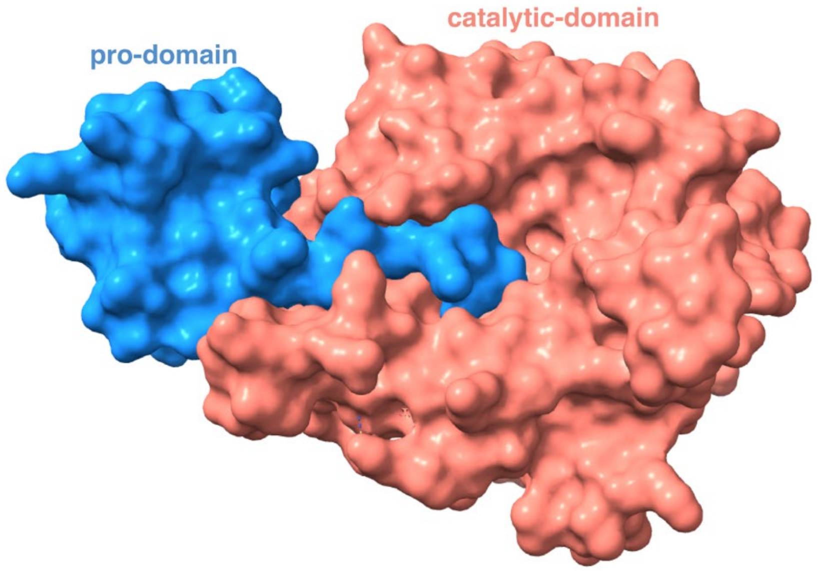

Supplementary Figure 12. Surface representation of CylA-96-412∘His_6-27-95 showing the extensive interactions between the pro-domain and the catalytic domain.

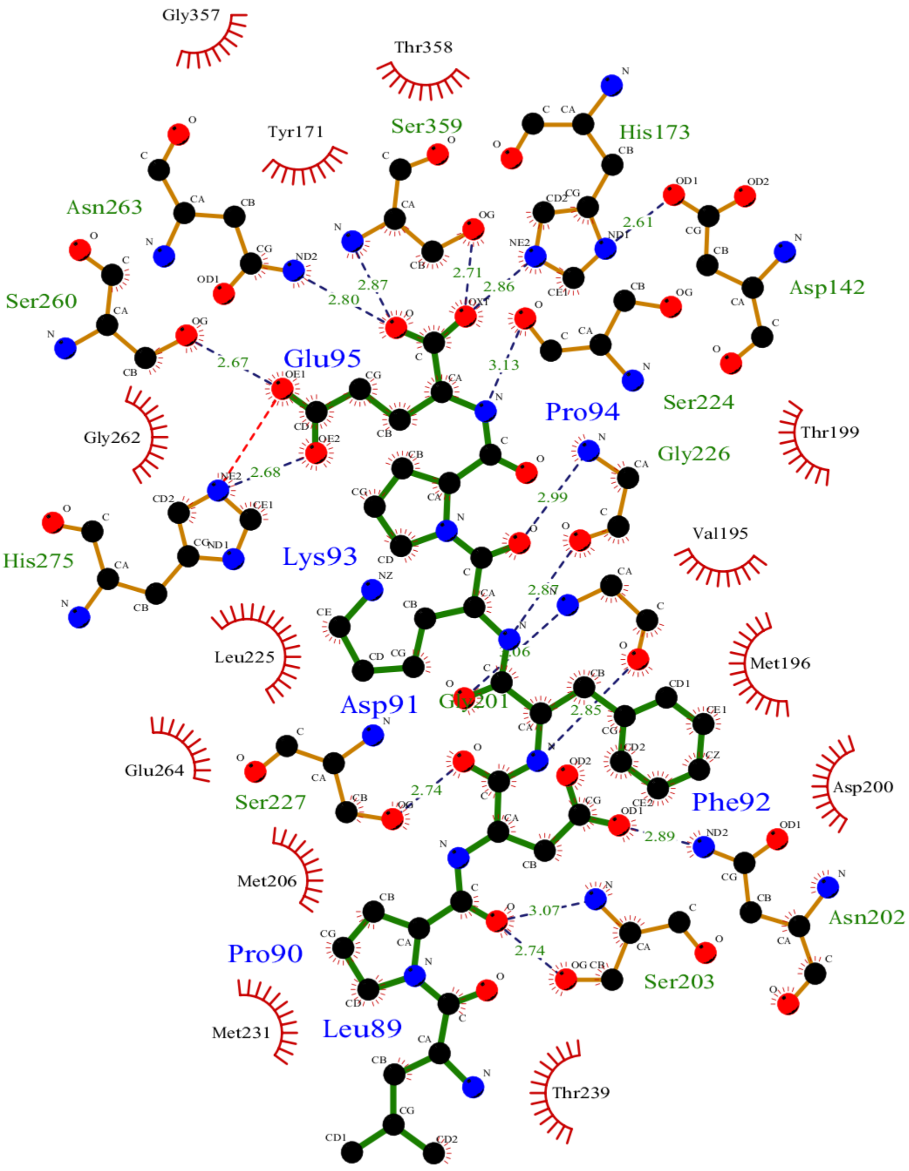

Supplementary Figure 13. Two-dimensional interaction diagram (generated using LigPlot+[3]) showing the interactions between the pro-domain cleavage peptide and active site residues in the catalytic domain.

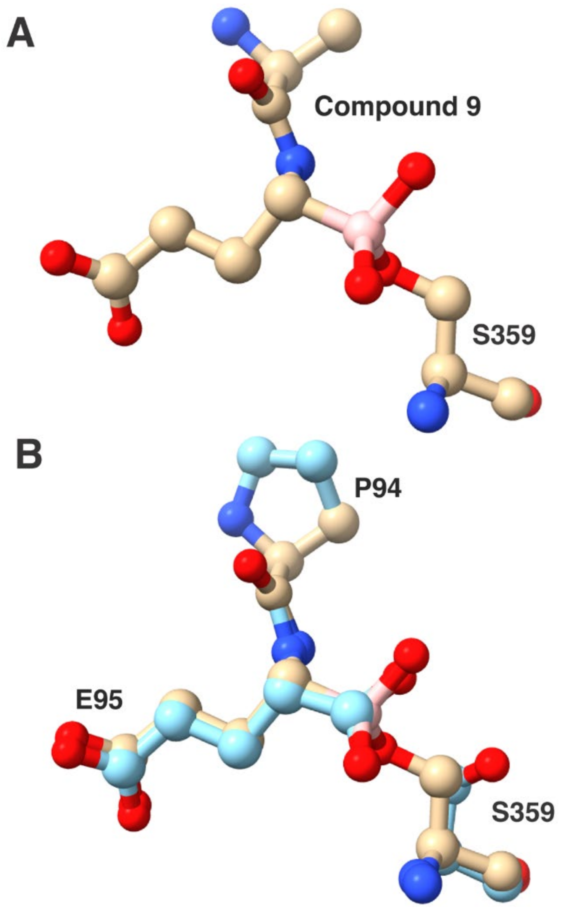

Supplementary Figure 14. A) Hypothetical model of compound **9** in a covalent complex with Ser359 of the CylA-96-412∘His_6-27-95 active site. The model of **9** is based on the position of the prodomain peptide observed in the experimental structure. B) Superposition of the hypothetical model (tan) with the experimentally determined CylA structure (cyan).

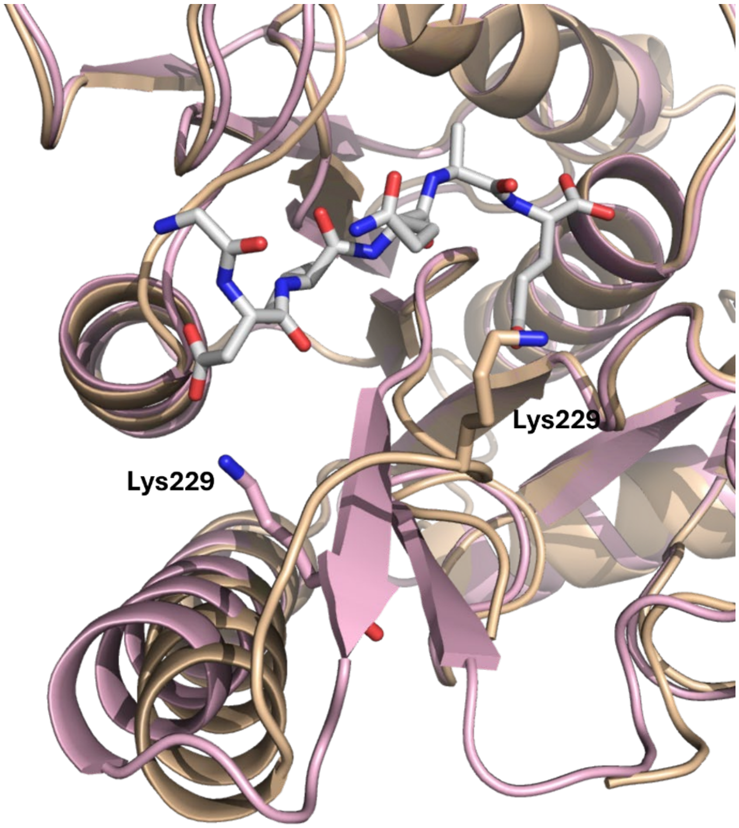

Supplementary Figure 15. Alignment of the C-terminal domain of CylA from the crystal structure (beige) with the same region from the AlphaFold 3 structure (light pink). The AlphaFold 3 structure was generated by asking AlphaFold 3 to generate a multimer composed of CylA-96-412 and GDVQAE. GDVQAE is shown as a stick structure (white). The side chain of Lys229 is made visible in each structure to show how its position may change during substrate binding.

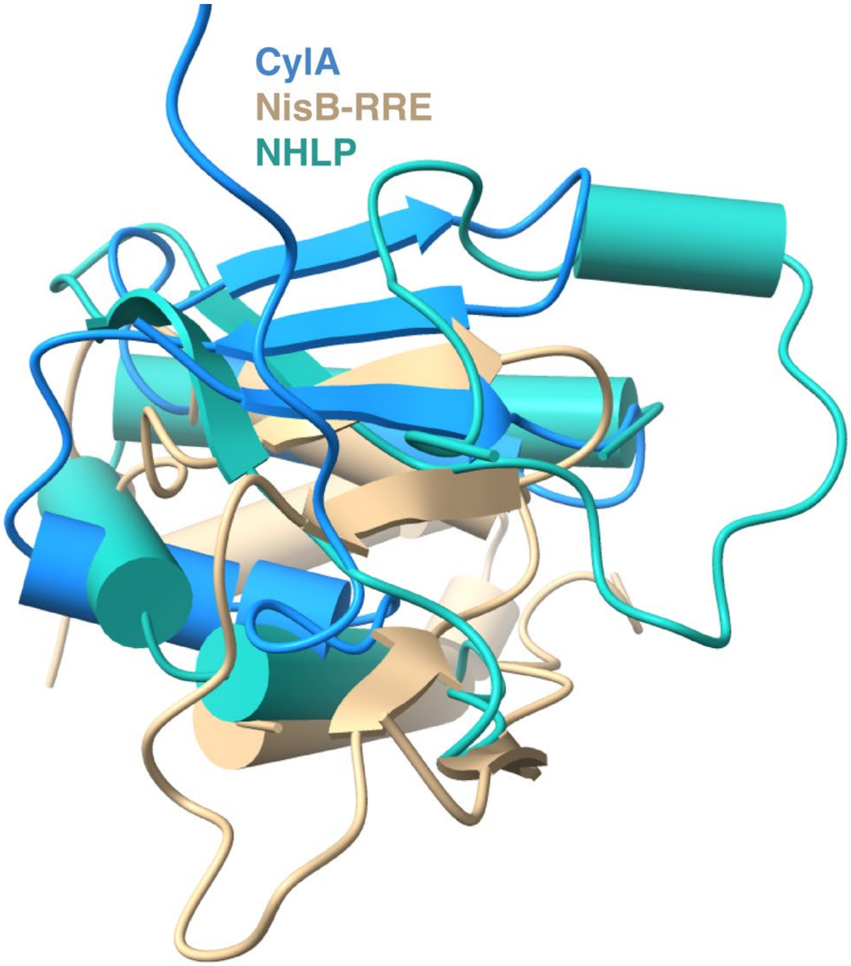

Supplementary Figure 16. Overlay of the pro-domain of CylA, the RRE of NisB and NHLP type LPs found in the proteusins (PDB = 8TB1)[4].

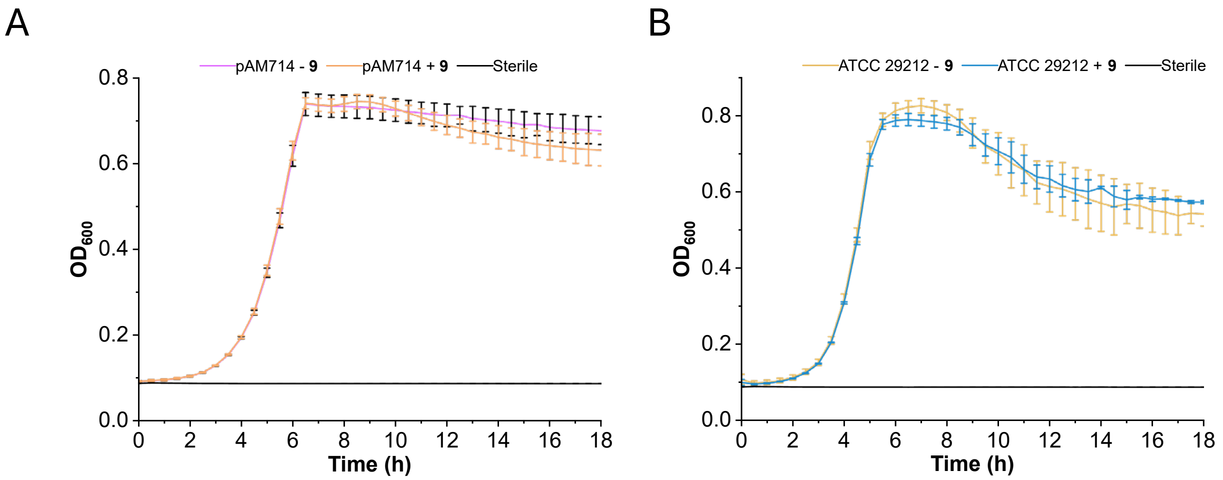

Supplementary Figure 17. Inhibitor **9** did not impact the growth of A) *E. faecalis* FA2-2 (pAM714) or B) *E. faecalis* ATCC 29212. Wells containing BHI media were inoculated with 1 x 10^6 CFU/mL of *E. faecalis*. Inhibitor **9** was added to achieve a well concentration of 100 μM. A growth control well not containing **9** and a sterile control not containing bacteria were set up on the same plate. The plate was incubated with shaking at 37℃ and the OD_{600} was measured every 30 min for 18h. This experiment was conducted on three biological samples (n = 3). Error bars represent the standard of deviation and are centered at the mean. Source data are provided as a Source Data file.

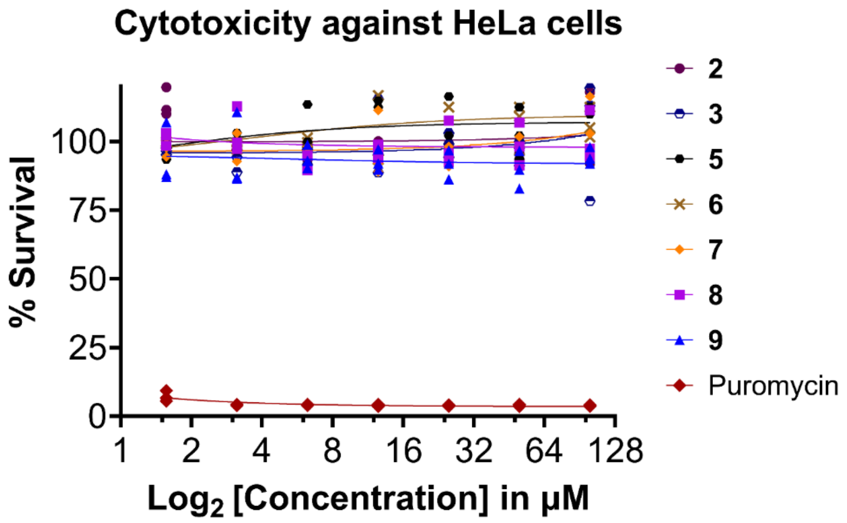

Supplementary Figure 18. Cytotoxicity assay of boronic acid inhibitors **2**, **3**, **5-9** against HeLa cells. Scatter plot shows that **2**, **3**, **5-9** do not display significant toxicity at concentration of 100 µM or less as determined by an MTT-based assay. Puromycin was used as positive control, while deionized sterile water was used as a negative control. Source data are provided as a Source Data file.

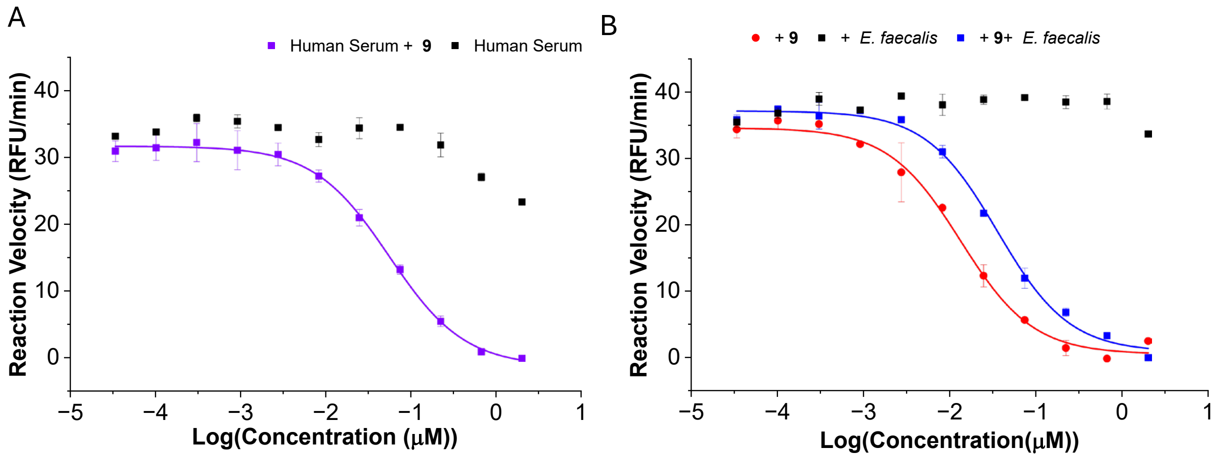

Supplementary Figure 19. The stability of inhibitor **9** in the presence of human serum or C+ *E. faecalis*. A) CylA inhibition activity of an extract of human serum treated with **9** (100 μM) for 3 h at 37 ℃. The resulting solution was used to determine the remaining activity of the inhibitor in a CylA activity assay. An IC_{50} equal to 55 ± 6 nM was determined. This experiment was performed on three different samples of inhibitor (n = 3). B) CylA inhibition activity of an extract of *E. faecalis* that was treated with **9** (100 μM) for 20 h at 37 ℃ (+**9** +*E. faecalis*). The resulting solution was used to determine the remaining activity of the inhibitor in a CylA activity assay. An IC_{50} equal to 36 ± 3 nM was determined. These values are higher than the IC_{50} for a fresh batch of inhibitor **9** (8.7 ± 0.9 nM, Fig. 5C) but the increase is relatively small and these data show compound **9** has relatively good stability in the presence of human serum and *E. faecalis*. This experiment was performed on two biological samples (n = 2). For A) and B) Error bars represent the standard of deviation centered at the mean. Non-linear concatenate fitting of data points v_i/v_o to a three-parameter function was used to determine IC_{50}. Source data are provided as a Source Data file.

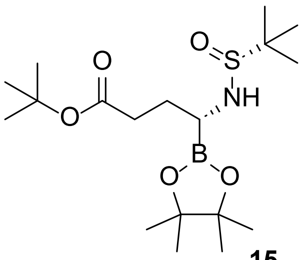

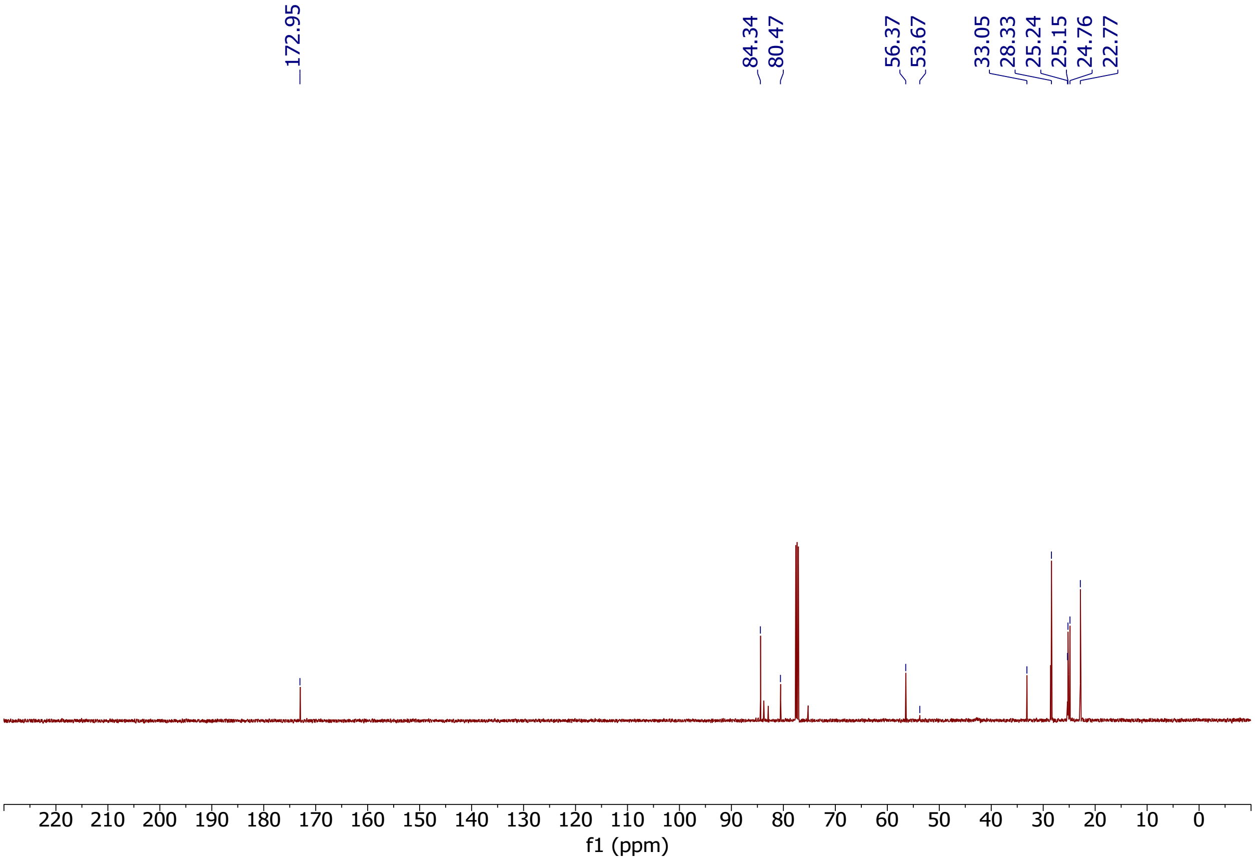

Supplementary Figure 20. ^{13}C NMR of compound **15**.

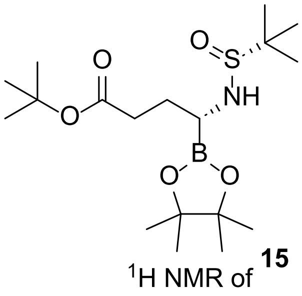

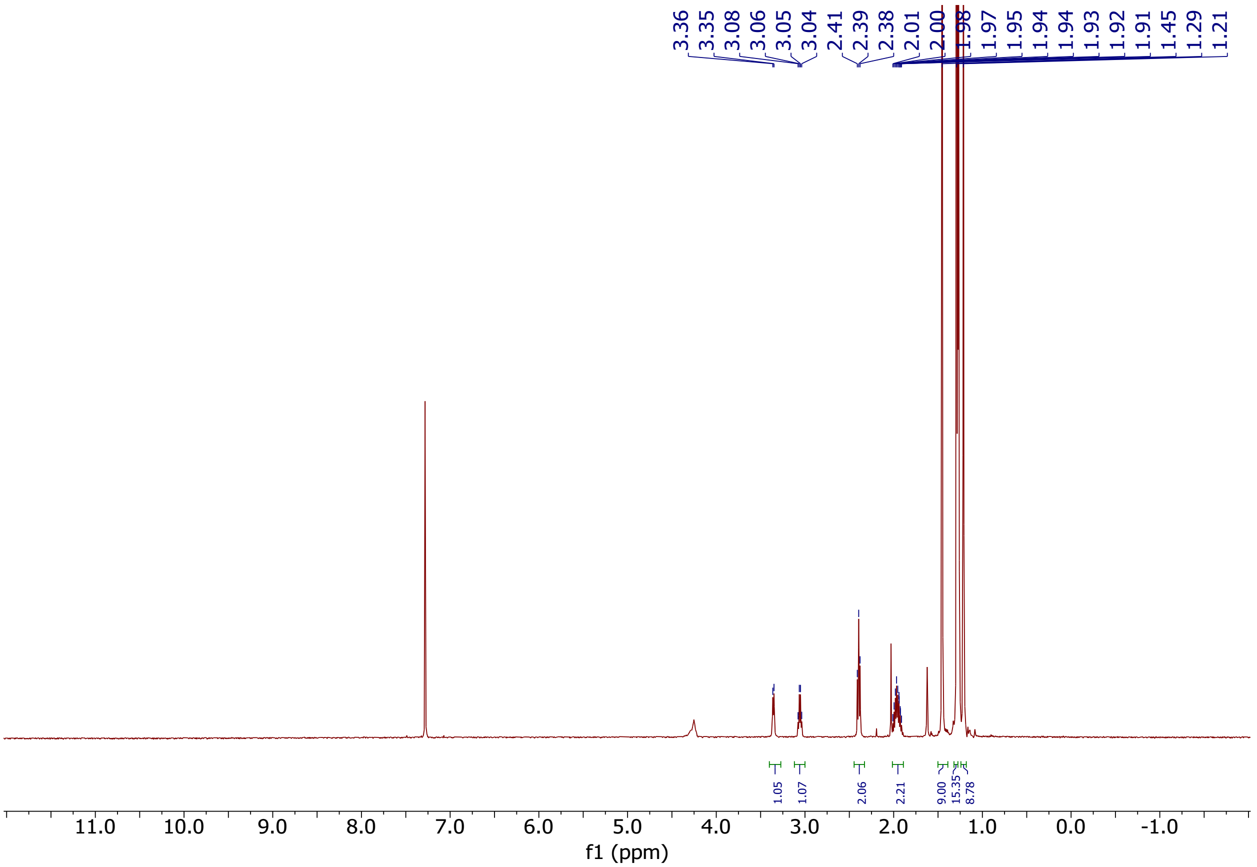

Supplementary Figure 21. ^{1}H NMR of compound **15**.

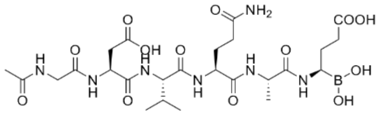

1

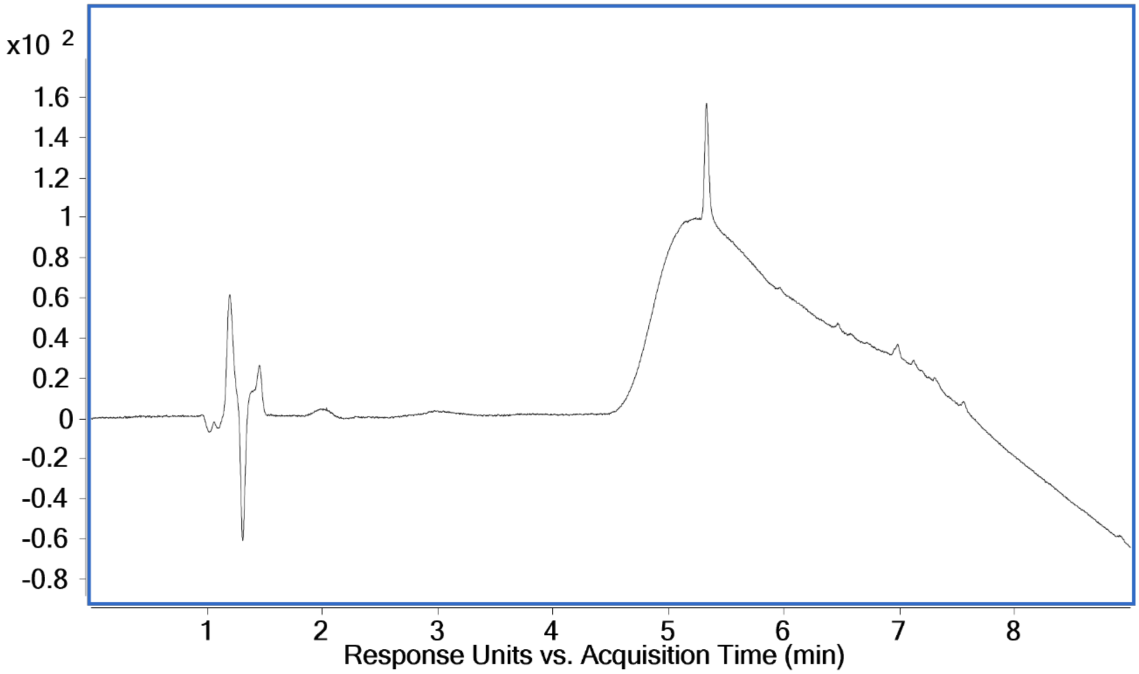
x10 2
1.6
1.4
1.2
1
0.8
0.6
0.4
0.2
0
-0.2
-0.4
-0.6
-0.8
1
2
3
4
5
6
7
8
Response Units vs. Acquisition Time (min)

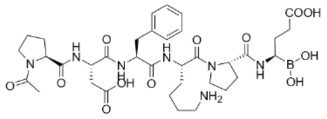

2

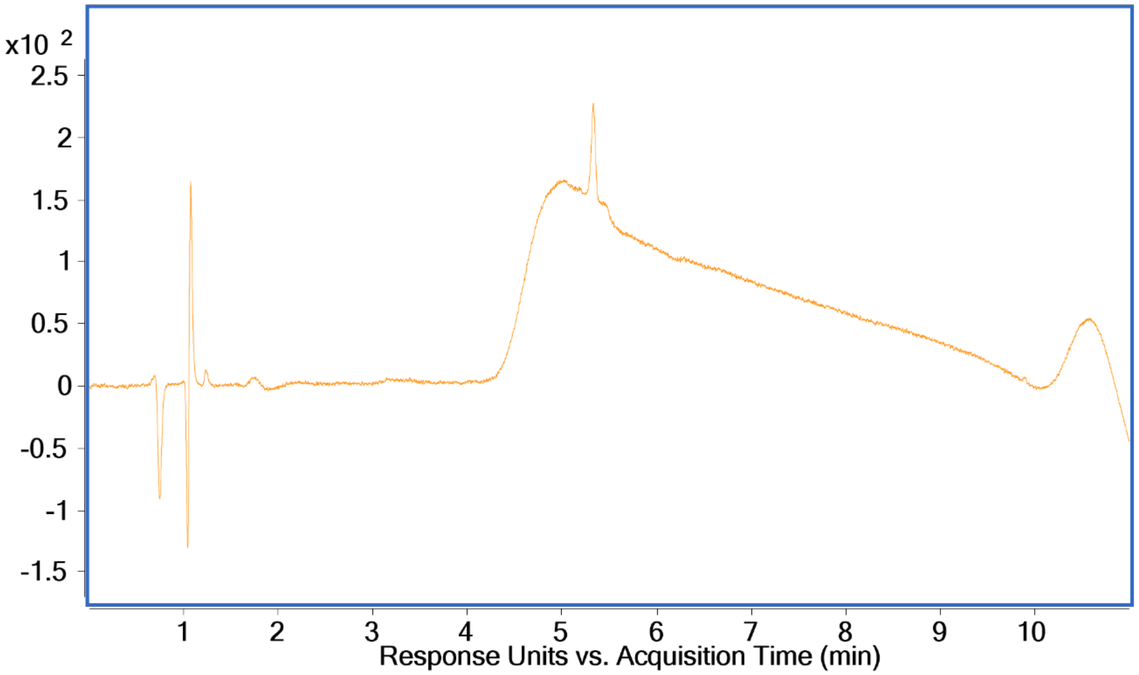
x10 2
2.5
2
1.5
1
0.5
0
-0.5
-1
-1.5
1
2
3
4
5
6
7
8
9
10
Response Units vs. Acquisition Time (min)

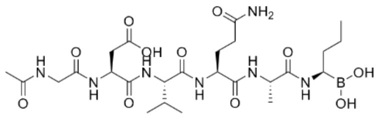
O
NH2
O
OH
H
N
O
O
H
N
H
N
O
H
N
O
N
H
B
OH
OH
O
N
H
O
O

3

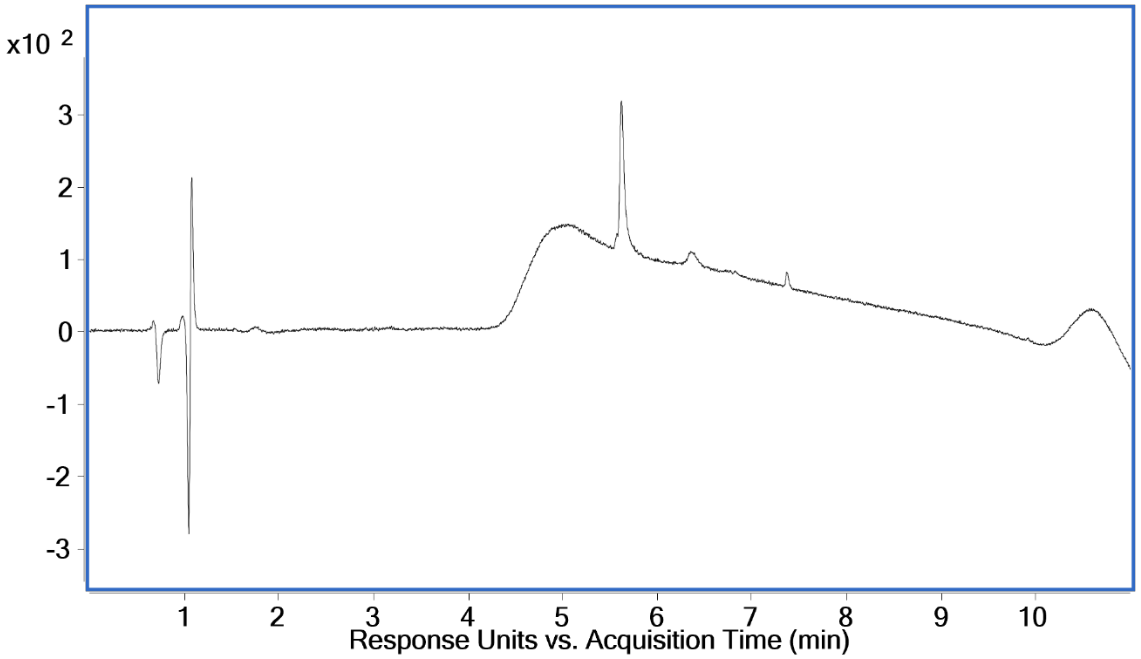
x10 2
3
2
1
0
-1
-2
-3
1
2
3
4
5
6
7
8
9
10
Response Units vs. Acquisition Time (min)

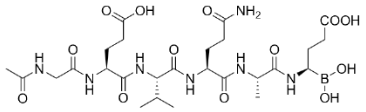
O
OH
O
NH2
COOH
H
N
O
N
H
O
H
N
O
N
H
O
H
N
O
N
H
B
OH
OH
O

4

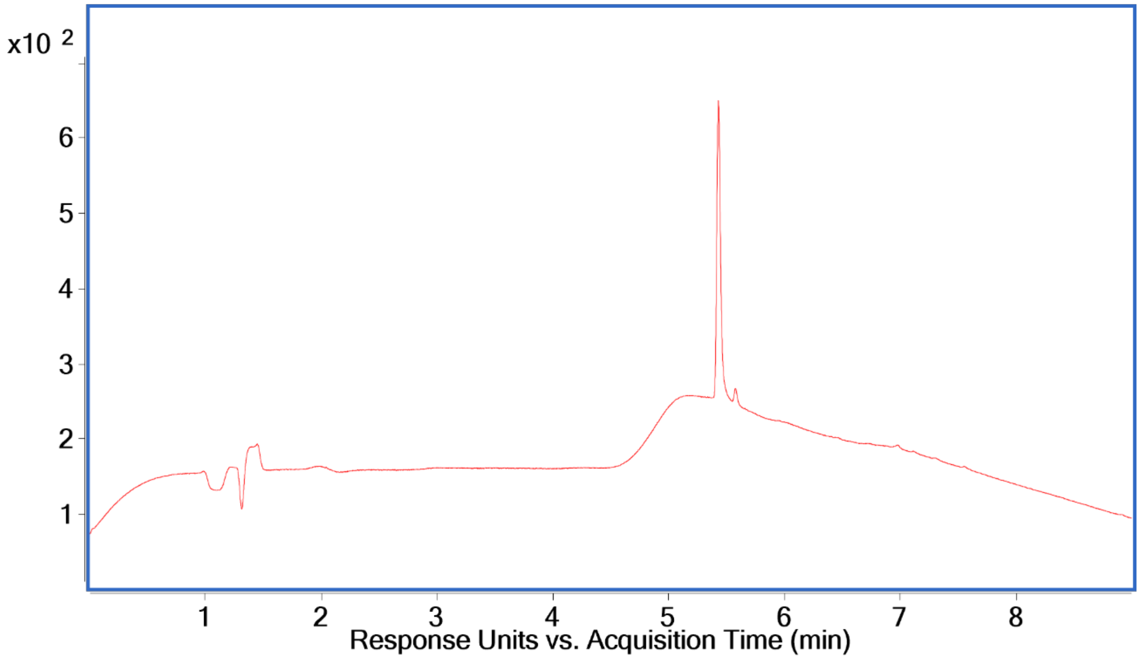
x10 2
6
5
4
3
2
1
1
2
3
4
5
6
7
8
Response Units vs. Acquisition Time (min)

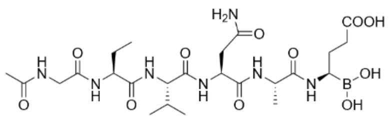
H2N
COOH
OH
B
OH

5

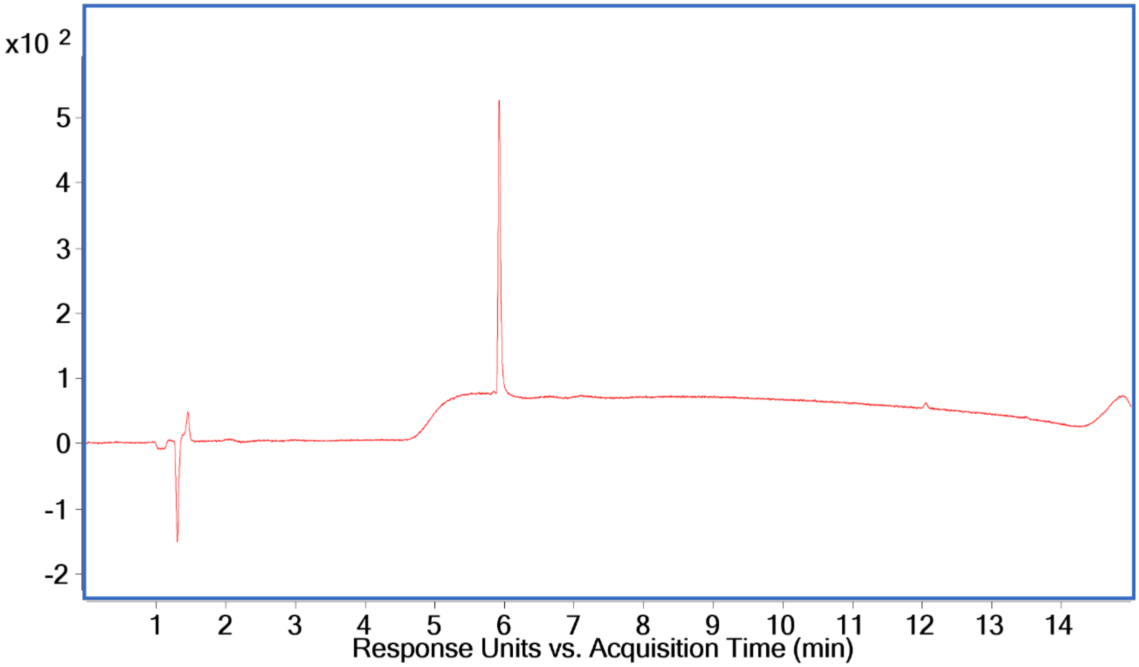
x10 2
5
4
3
2
1
0
-1
-2
1
2
3
4
5
6
7
8
9
10
11
12
13
14
Response Units vs. Acquisition Time (min)

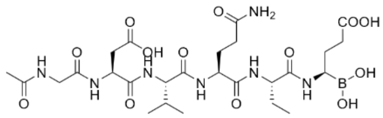
NH2
COOH
OH
B
OH

6

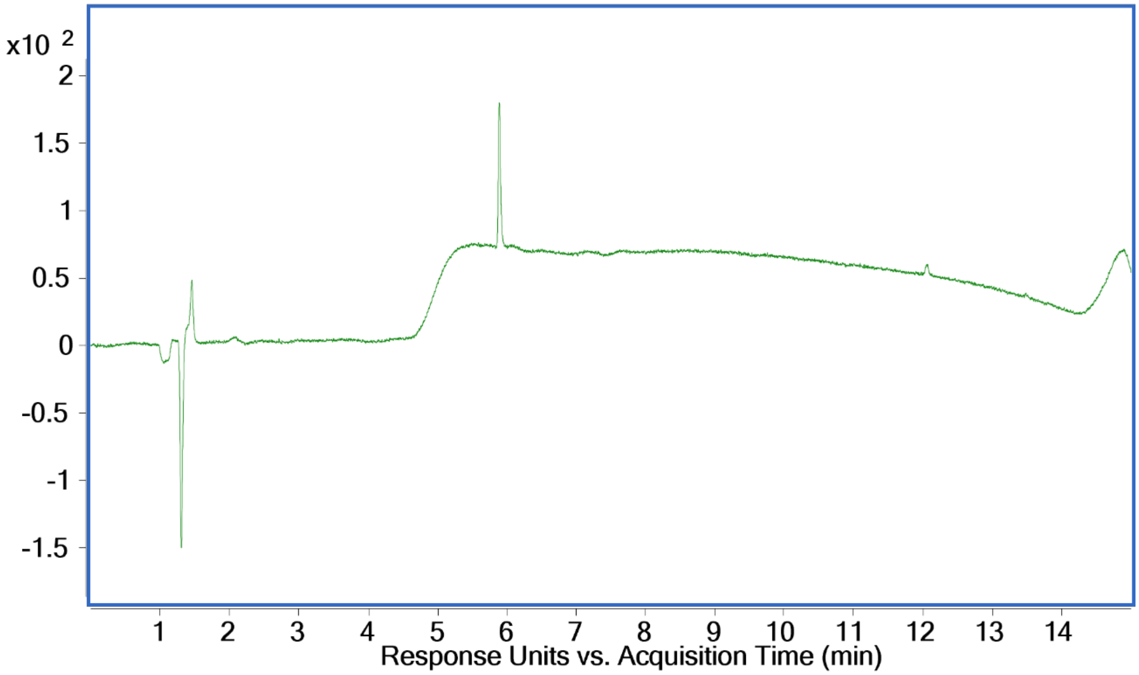
x10 2
2
1.5
1
0.5
0
-0.5
-1
-1.5
1
2
3
4
5
6
7
8
9
10
11
12
13
14
Response Units vs. Acquisition Time (min)

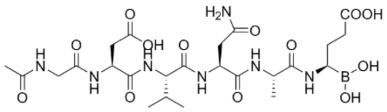

7

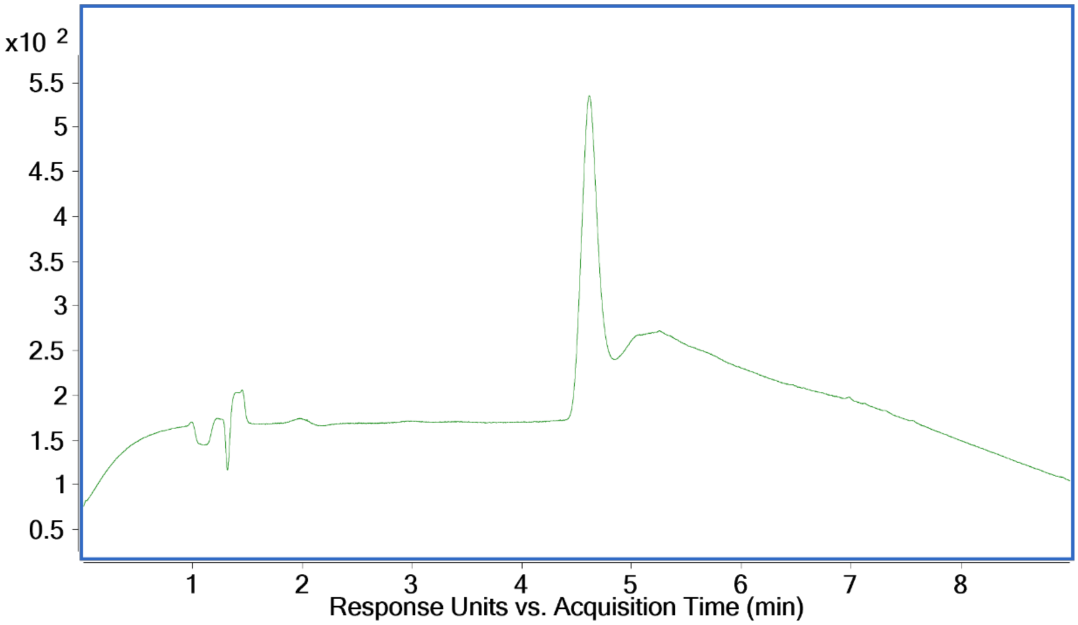

x10 2
Response Units vs. Acquisition Time (min)

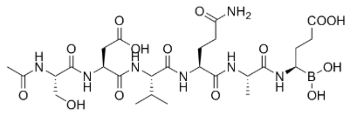

8

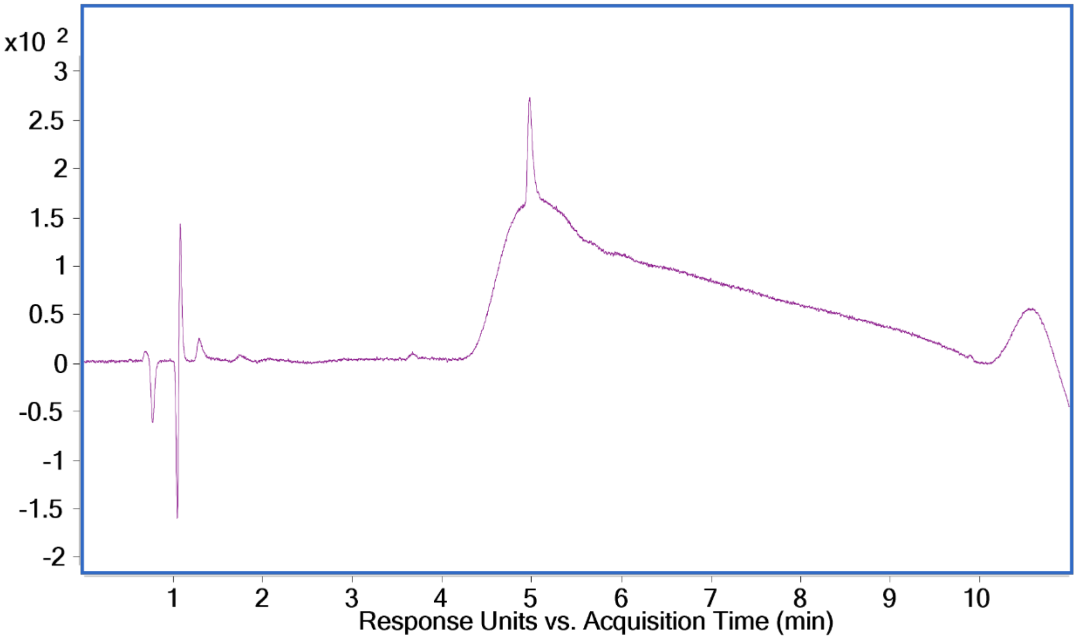

x10 2
Response Units vs. Acquisition Time (min)

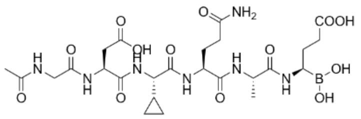

9

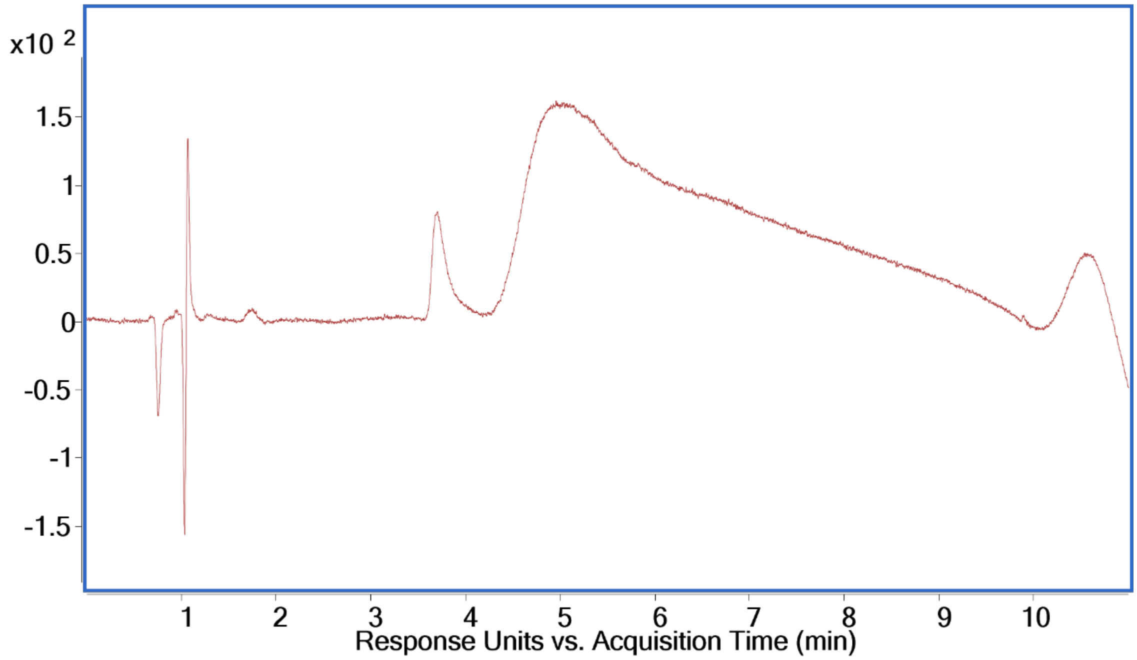
x10 2
1.5
1
0.5
0
-0.5
-1
-1.5
1 2 3 4 5 6 7 8 9 10
Response Units vs. Acquisition Time (min)

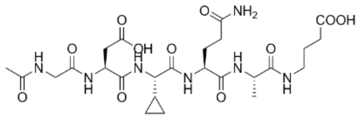

10

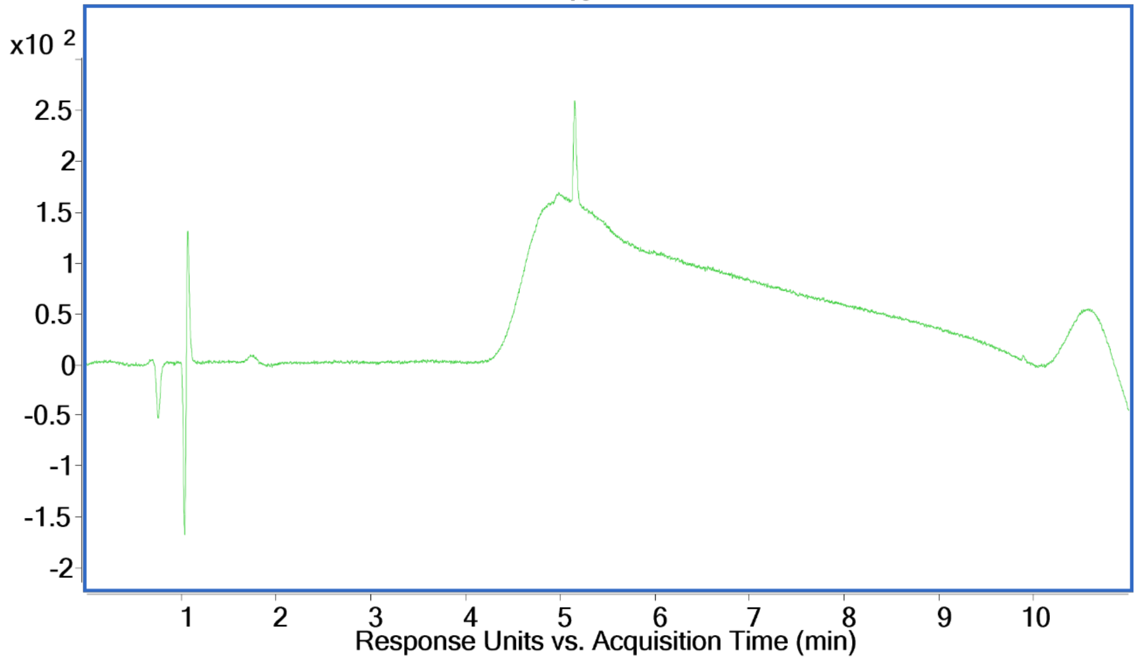
x10 2
2.5
2
1.5
1
0.5
0
-0.5
-1
-1.5
-2
1 2 3 4 5 6 7 8 9 10
Response Units vs. Acquisition Time (min)

Supplementary Figure 22. Analytical UPLC traces of **1-10** collected using a UV-Vis detector set to an absorbance of 205 nm. Traces were collected using an Agilent 1290 LC-MS QToF instrument equipped with a Poroshell C18 2.7 µm 120 Å 100 x 3.0 mm column. The following LC method was used for separation: 98% H_2O + 0.1% formic acid (FA, solvent A)/2% CH_3CN + 0.1% FA (solvent B) for 2 min then a linear gradient from 98:2 to 2:98 solvent A:solvent B over 6 min. Source data is included in the Source Data file.

References

1 Brademan, D. R., Riley, N. M., Kwiecien, N. W. & Coon, J. J. Interactive peptide spectral annotator: A versatile web-based tool for proteomic applications. *Mol. Cell. Proteom.* **18**, S193-S201 (2019).

2 Abramson, J. *et al.* Accurate structure prediction of biomolecular interactions with AlphaFold 3. *Nature* **630**, 493-500 (2024).

3 Laskowski, R. A. & Swindells, M. B. LigPlot+: multiple ligand-protein interaction diagrams for drug discovery. *J. Chem. Inf. Model.* **51**, 2778-2786 (2011).

4 Nguyen, N. A. *et al.* Disordered regions in proteusin peptides guide post-translational modification by a flavin-dependent RiPP brominase. *Nat. Comm.* **15**, 1265 (2024).
